# SimPLIT: Simplified Sample Preparation for Large-Scale Isobaric Tagging Proteomics

**DOI:** 10.1021/acs.jproteome.2c00092

**Published:** 2022-07-18

**Authors:** Fernando J. Sialana, Theodoros I. Roumeliotis, Habib Bouguenina, Laura Chan Wah Hak, Hannah Wang, John Caldwell, Ian Collins, Rajesh Chopra, Jyoti S. Choudhary

**Affiliations:** † Functional Proteomics Group, The Institute of Cancer Research, Chester Beatty Laboratories, London SW3 6JB, U.K.; ‡ Cancer Research UK Cancer Therapeutics Unit, The Institute of Cancer Research, London SM2 5NG, U.K.

**Keywords:** isobaric labeling, TMTpro, cancer cell lines, targeted protein degradation, IMiDs, CELMoDs

## Abstract

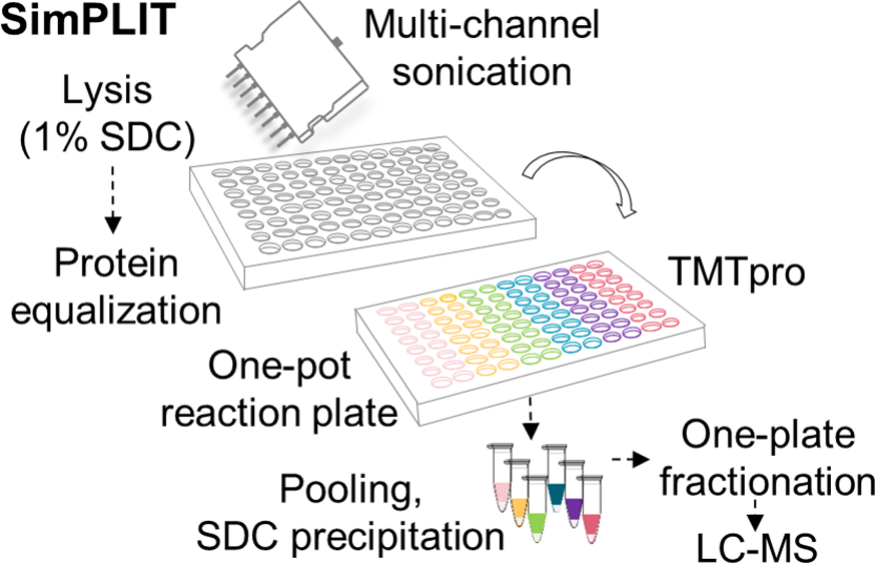

Large scale proteomic profiling of cell lines can reveal molecular signatures attributed to variable genotypes or induced perturbations, enabling proteogenomic associations and elucidation of pharmacological mechanisms of action. Although isobaric labeling has increased the throughput of proteomic analysis, the commonly used sample preparation workflows often require time-consuming steps and costly consumables, limiting their suitability for large scale studies. Here, we present a simplified and cost-effective one-pot reaction workflow in a 96-well plate format (SimPLIT) that minimizes processing steps and demonstrates improved reproducibility compared to alternative approaches. The workflow is based on a sodium deoxycholate lysis buffer and a single detergent cleanup step after peptide labeling, followed by quick off-line fractionation and MS2 analysis. We showcase the applicability of the workflow in a panel of colorectal cancer cell lines and by performing target discovery for a set of molecular glue degraders in different cell lines, in a 96-sample assay. Using this workflow, we report frequently dysregulated proteins in colorectal cancer cells and uncover cell-dependent protein degradation profiles of seven cereblon E3 ligase modulators (CRL4^CRBN^). Overall, SimPLIT is a robust method that can be easily implemented in any proteomics laboratory for medium-to-large scale TMT-based studies for deep profiling of cell lines.

## Introduction

Multiplexed protein quantification using mass spectrometry coupled with isobaric peptide labeling has enabled the simultaneous comparison of multiple proteomes with high reproducibility and minimum missing values. In combination with extensive multidimensional peptide separation, proteomic analysis with genome-wide coverage and high quantitation accuracy has been feasible, providing a powerful tool to investigate complex and dynamic biological systems.

Despite the great advancements in proteomics technologies, there is a growing demand for high throughput and large-scale proteomic data acquisition to support biomarker and drug target discovery applications.^1^ In this regard, the synthesis of tandem mass tags with increased multiplexing capabilities (TMTpro, up to 18-plex) has greatly facilitated high-throughput deep proteomics analysis by reducing the number of peptide fractionation sets required within a study and by minimizing LC-MS machine usage time.^2,3^ Typically, depending on the sample complexity, a deep multiplexed proteomics experiment is acquired in 3–5 days from sample preparation to mass spectrometry analysis, which is often a limiting factor for the design of large-scale studies. Bottlenecks in workflows have been addressed through the development of approaches that encompass efficient sample preparation, enhanced peptide separation,^4^ high sensitivity mass spectrometry,^5^ and fast real-time data processing.^6,7^ Specifically, mass spectrometry instruments with real-time database search capabilities can reduce MS analysis time while maintaining high accuracy and precision.^6,8^ Widely used sample preparation methods that utilize detergents (e.g., SDS) or chaotropes (e.g., urea) in lysis buffers to efficiently solubilize proteins have also delivered proteomes quantitated in-depth.^9,10^ However, such workflows require complex protein and/or peptide cleanup steps to eliminate buffer components that are not compatible with proteases, chemical labeling reagents, and/or MS acquisition. Clean-up strategies include protein precipitation (acid or organic solvents),^11^ aggregation or trapping of proteins in beads/resins (SP3, S-TRAP, iST),^12−14^ using centrifugation filtration columns (FASP),^15^ and solid-phase C18 extraction-evaporation.^3^ Adapting these workflows for large scale experiments has several limitations, as complex multistep processing of samples is prone to random errors leading to higher sample-to-sample variability, in addition to being more laborious.

Several strategies have been reported to streamline TMT sample preparation with the aim of minimizing processing steps and reducing individual sample variability. For example, samples digested on-pellet are compatible with cleanup steps after the TMT-labeled peptides have been combined.^11^ Similarly, we and others have described a single detergent removal step by acid precipitation or phase transfer after digestion and isobaric labeling using sodium deoxycholate (SDC)-based lysis buffers.^16−18^ To facilitate the processing of laborious multistep cleanup (AutoSP3) or the dispensing of reagents (digestion and TMT labeling), liquid handling platforms in 96-well formats have also been described (AutoMP3, nanoPOTs) for large-scale studies.^8,19,20^ TMT based high-throughput proteome profiling with minimal sample processing has also been very successful in the field of single-cell proteomics.^21,22^ Further optimization and simplification of sample preparation workflows from lysate generation to sample injection could offer important benefits for large scale bulk cell sample preparation.

Herein, we set out to develop a TMT-based sample preparation and analysis workflow with the smallest possible number of steps that can be easily applied in multiple batches of samples in a 96-well plate without the use of additional costly reagents or specialized expensive equipment, while maintaining high reproducibility and depth of proteome analysis. We provide a reliable and less laborious workflow that can be quickly adopted by any proteomics lab for medium-to-large scale TMT-based studies involving the analysis of cell lines. Our simplified workflow (SimPLIT) relies on the use of an SDC-based lysis buffer and one-pot successive reactions with parallel processing followed by quick off-line fractionation and MS2 analysis. To evaluate the performance of the SDC-based lysis buffer for TMT preparation, we performed a comparison against commonly used TMT sample preparation approaches for the analysis of cell lines in a multiplexed experiment, which showed the suitability of our workflow for fast, reproducible, and deep proteome analysis. We demonstrate applicability of the SimPLIT platform for large-scale quantitation on two use cases: first, for cell line characterization by acquiring 48 proteomes of a colorectal adenocarcinoma (COREAD) panel, and second, analysis of 48 proteomes from drug-treated cell lines. Proteomics data from the COREAD proteomes have been previously published from our lab,^23^ providing a benchmarking of the workflow and highlighting a robust subset of frequently dysregulated proteins as well as proteins associated with microsatellite instability (MSI). The drug-treatment proteomes provide insight on the drug-induced protein target landscape of seven clinical cereblon molecular glue degraders in three cell lines from different tissue origins.

In summary, we demonstrate that our simplified large-scale workflow for TMT-proteomics, which can be easily implemented in any proteomics laboratory, is highly reproducible with a low method-associated bias when compared to alternative approaches.

## Experimental Procedures

### Comparison of the Different Methods

A detailed description of the multiplexed comparison of the different methods is provided in the Supporting Information.

### Compounds, Cell Lines, and Antibodies

#### Compounds

Lenalidomide (Abcam), pomalidomide (Abcam), Avadomide/CC-122, (Aquila Pharmatech), Iberdomide/CC-220 (MedChem Express), Mezigdomide/CC-92480 (WuXi AppTec), CC-90009 (WuXi AppTec), CC-885 (MedChem Express), and MLN4924 were purchased from the indicated suppliers and were subjected to in-house LC-MS for quality control.

#### Antibodies

Primary and secondary antibodies used included anti-IKZF1 at 1:1000 dilution (Cell signaling, #14859), anti-IKZF2 at 1:1000 dilution (Cell Signaling, #42427), anti-GSPT1 at 1:2000 dilution (Sigma, hpa052488), anti-Actin at 1:5000 dilution (Abcam, ab8226), IRDye 680LT Goat anti-Mouse IgG (Licor, 926-68020), and IRDye 800CW Goat anti-Rabbit IgG at 1:5000 dilution (Licor, 926-32211) were used as secondary antibodies.

### Cancer Cell Line Culture and Treatment

#### Cell Line Culture

MM1S and HL60 cell lines were passaged in RPMI media supplemented with 10% FBS, and HCT116 cell lines were grown in DMEM media supplemented with sodium pyruvate and 10% FBS. For the colorectal cancer cell lines, we used replicate cell pellets that were collected during a previous study of our lab and were stored at −80 °C.^23^

#### Cellular Activity of Compounds in MM1S, HL60, and HCT116

DMSO-solubilized compounds were dispensed into inverted microplates (Corning, 3701) to cover a 12-point dilution range with 3-fold increments, starting from 50 μM. 8000 MM1S cells and 2000 HL60 cells in 40 μL of RPMI media were seeded onto the predispensed microplates and incubated for 5 days at 37 °C and 5% CO_2_. In the case of HCT116 cells, 1000 cells were seeded in DMEM media the day before compound dispensing. After a 5-day incubation, cell viability was measured. Five μL of Cell Titer (Promega) was added per well, and the absorbance was recorded after a 3-h incubation at 37 °C using the EnVision Multimode Plate Reader (PerkinElmer). Each plate was first normalized against the positive and negative controls, and the Z′-factors were then used to control the quality of each plate. Data were plotted as percent inhibition of viability versus drug concentration and were fitted using four-parameter dose–response curves (GraphPad Prism). A compound was annotated as active when the IC_50_ < 10 μM and max kill of greater than 50% after a 5-day exposure to a cell line.

### Quantitative Mass Spectrometry-Based Proteomics for SimPLIT

#### Optimized Sample Preparation for 96 Samples Using SimPLIT

Frozen pellets of ∼2 × 10^6^ cells were suspended in 70 μL lysis buffer consisting of 1% sodium deoxycholate (SDC), 100 mM triethylammonium bicarbonate (TEAB), 10% isopropanol, 50 mM NaCl, supplemented with protease and phosphatase inhibitor cocktail (Thermo, Halt, #78429) and transferred into PCR eight-tube strips (0.2 mL) fitted into a 96-well plate rack (Eppendorf #30124359). Alternatively, common PCR plates (Eppendorf #30129504) can be used; however, here we used 8-strip PCR tubes that have individual caps and can be useful during sample handling and heating steps. Additionally, the use of 8-strip PCR tubes can allow direct collection and washing of the cell pellets. Homogenization was carried out using an 8-tip horn sonication probe (Fisherbrand, #12357338) for 2 × 30 s with pulses of 1 s at 40% amplitude (EpiShear). The 8-tip horn enables processing of 8 samples simultaneously in a standard 8-strip PCR tube or a 96-well plate. The sonication steps were performed in multiple short pulses while keeping the samples in a cooler rack at 0 °C that changes color when the temperature has exceeded 7 °C. After sonication, all samples were diluted with an additional 70 μL of lysis buffer, resuspended, and heated at 90 °C for 5 min. Protein concentration was measured with the Rapid Gold BCA Protein Assay (Pierce, #15776178) according to the manufacturer’s instructions, and sample concentrations were equalized by the addition of lysis buffer.

Equal aliquots containing 30 μg of total protein were transferred into a clean 96-well PCR plate (Eppendorf #30129504 or #30124359) for further processing. The cysteines were reduced with 5 mM tris-2-carboxyethyl phosphine (TCEP, #10657344, Thermo Scientific) for 1 h at 60 °C and alkylated by 10 mM iodoacetamide (IAA) for 30 min at room temperature in the dark, with reagents transferred with a multichannel pipet. For proteolytic digestion, 6 μL of trypsin stock solution (500 ng/μL in 0.1% formic acid, Pierce, #90059) were added to each sample and incubated for 2 h at 37 °C, then at RT overnight in a shaker at 600 rpm. After trypsin digestion, samples were dried in SpeedVac concentrator with a well-plate rotor and reconstituted in 25 μL of 100 mM TEAB prior to labeling. Digested samples were labeled with 10 μL of TMTpro-16plex aliquots in extra dry acetonitrile (TMTpro: 25 μg/μL, Thermo Scientific). The TMT reagents (5 mg vials) were reconstituted and aliquoted in 8-strip PCR tubes to enable the use of a multichannel pipet for the labeling step. Hydroxylamine was used to quench the reaction, and then all TMT labeled samples of the same batch were combined into a single tube. The TMT peptide mixture was acidified with 1% formic acid, and the precipitated SDC was removed by centrifugation at 10 000 rpm for 5 min. The supernatant was dried with a centrifugal vacuum concentrator.

#### High pH Reversed-Phase Peptide Fractionation for SimPLIT

Offline peptide fractionation was based on high pH reversed-phase (RP) chromatography using the Waters XBridge C18 column (2.1 × 150 mm, 3.5 μm) on a Dionex UltiMate 3000 HPLC system at a flow rate of 0.2 mL/min. Mobile phase A was 0.1% (v/v) ammonium hydroxide, and mobile phase B was acetonitrile, 0.1% (v/v) ammonium hydroxide. Pooled TMT-peptides were resuspended in 200 μL of buffer A, centrifuged at 14 000 rpm for 5 min, and the supernatant was injected for fractionation with the following gradient: isocratic for 5 min at 5% phase B, gradient for 40 min to 35% phase B, gradient to 80% phase B in 5 min, isocratic for 5 min, and re-equilibrated to 5% phase B. For the SimPLIT experiments, 12 retention time-based fractions were collected into a deep 96-well plate (Waters, #186009184) and SpeedVac dried. The peptides were then resuspended in the well plate with 0.1% formic acid and finally pooled into eight fractions by combining the first and the last four fractions. All six TMT-16plex sets were fractionated, evaporated, resuspended, and injected for LC-MS using the same deep 96-well plate.

#### LC-MS Analysis for SimPLIT

LC-MS analysis was performed on the Dionex UltiMate 3000 UHPLC system coupled with the LTQ Orbitrap Lumos mass spectrometer (Thermo Scientific). Samples were analyzed with the EASY-Spray C18 capillary column (75 μm × 50 cm, 2 μm) at 50 °C. Mobile phase A was 0.1% formic acid and mobile phase B was 80% acetonitrile, 0.1% formic acid. The gradient separation method was as follows: 150 min gradient up to 38% B, for 10 min up to 95% B, for 5 min isocratic at 95% B, re-equilibration to 5% B in 10 min, for 10 min isocratic at 5% B.

Precursors between 375 and 1500 *m*/*z* were selected with a mass resolution of 120 000, automatic gain control (AGC) of 4 × 10^5^, and IT (injection time) of 50 ms, with the top speed mode in 3 s for high collision dissociation (HCD) fragmentation with a quadrupole isolation width of 0.7 Th (Thomson unit). The collision energy was set at 35%, with AGC at 1 × 10^5^ and IT at 86 ms. The HCD MS2 spectra were acquired with a fixed first mass at 100 *m*/*z* and a resolution of 50 000. Targeted precursors were dynamically excluded for further isolation and activation for 45 s with 7 ppm mass tolerance.

#### Database Search and Protein Quantification for the SimPLIT Method

The SEQUEST-HT search engine was used to analyze the acquired mass spectra in Proteome Discoverer 2.4 (Thermo Scientific) for protein identification and quantification. The precursor mass tolerance was set at 20 ppm, and the fragment ion mass tolerance was set at 0.02 Da. Spectra were searched for fully tryptic peptides with a maximum of 2 missed-cleavages. TMTpro on lysine residues and peptide N-termini (+304.2071 Da) and carbamidomethylation of cysteine residues (+57.0215 Da) were set as static modifications, while oxidation of methionine residues (+15.9949 Da) and deamidation of asparagine and glutamine (+0.9848 Da) were set as variable modifications.

Peptide confidence was estimated with the Percolator node. Peptides were filtered at *q*-value <0.01 based on a decoy database search. All spectra were searched against UniProt-SwissProt proteomes of reviewed *Homo sapiens* protein entries (version 12-June-2020) appended with contaminants and FBS proteins.^24^ The reporter ion quantifier node included a TMTpro quantification method with an integration window tolerance of 15 ppm and an integration method based on the most confident centroid peak at the MS2 level. Only unique peptides were used for quantification, with protein groups considered for peptide uniqueness. Peptides with an average reporter signal-to-noise ratio >3 were used for protein quantification. Correction for the isotopic impurity of reporter quantification values was applied.

Peptide TMTpro signal-to-noise (S/N) values were normalized to the sum per channel. For each protein, normalized peptide S/N values were summed to create protein quantification values. The data were scaled per protein such that the average of all samples within a set is 100. Protein ratios were directly calculated from the grouped protein abundances. No imputation for missing values was performed.

### Experimental Design and Statistical Rationale

#### Proteomics Data Normalization, Analysis, and Visualization

Quantitative and statistical methods of analysis for all the experiments are described in the Results, figure legends, and Supporting Information sections. The web-based tool Phantasus^25^ was used for generating similarity matrices, hierarchical clustering, and visualization of heatmaps. Protein annotations were obtained from KEGG, GSEA, and UniProt.^26−28^ Additional visualization was performed in GraphPad Prism (ver. 9.1.2) and Cytoscape.^29^ Annotation enrichment was performed in Perseus.^30^ For significant protein enrichments, one-sample *t* tests and Welch’s *t* test were performed in Perseus.^30^

#### Methods Comparison Proteomics Data Set

To compare the performance of the different proteomic methods, the deviation-from-average ratios were determined by calculating sample protein abundance differences from the average of all methods (sample/average, log_2_). Protein size and the number of transmembrane domain annotations were obtained from UniProt.^28^ Boxplots were generated in Graphpad Prism (ver. 9.1.2).

#### COREAD Proteomics Data Set

Clustered pairwise correlation matrices were generated using the scaled protein abundances of the data sets followed by unsupervised hierarchical clustering as implemented and visualized in Phantasus.^25^ To combine the quantitative data, the scaled protein abundances were log_2_ transformed and centered to zero followed by column Z-score normalization. For significant protein enrichments, one-sample *t* tests, and Welch’s *t* test were performed in Perseus.

#### Drug-Treated Proteomics Data

Significant changes between DMSO and compound treatment were assessed with a two-sample *t* test as implemented in Proteome Discoverer 2.4. Significant targets (*P* < 0.001 and log_2_FC < −0.32) with at least two unique peptides are reported.

## Results

### Evaluation of a Simplified Sample Preparation Protocol for Isobaric Labeling Proteomics

To develop a simplified sample preparation proteomics workflow, we leveraged the compatibility of sodium deoxycholate (SDC) based lysis buffers with trypsin and TMT-reagents, which enables the use of the same buffer from cell lysis to isobaric labeling. Moreover, as dilutions or removal of detergents prior to tryptic digestion are circumvented, a low digestion volume is maintained, enabling direct addition of TMT reagents for labeling. Together these advantages allow the implementation of a simplified one-pot preparation, through successive addition of reagents onto a single vessel. The workflow only requires a single detergent removal step by acid precipitation after combining the TMT-labeled peptides. Performing acid precipitation on the TMT-peptide mixture just after the reaction should be less susceptible to aggregation/adhesion effects as it contains organic solvents from which the peptides are better recovered. Altogether, the reduced sample handling between protein assay and peptide fractionation steps minimizes the overall sample-to-sample processing variation.

**Figure 1:**
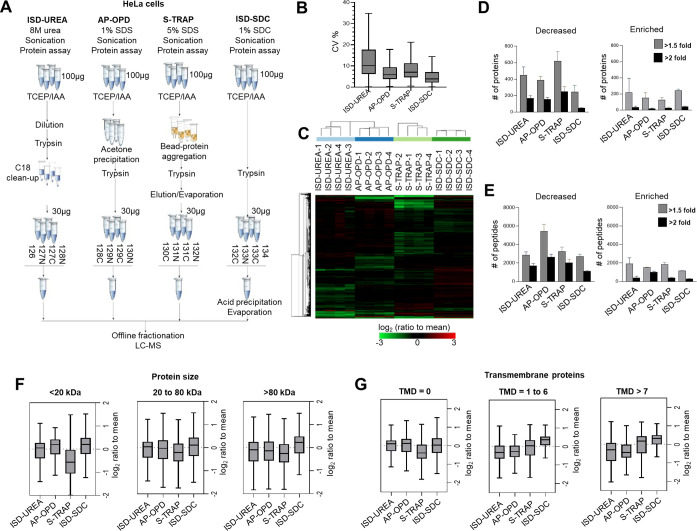
Quantitative comparison of four proteomic sample preparation workflows. (A) Experimental workflow for multiplexed proteomics comparison of in-solution digestion with urea (ISD-UREA), acetone precipitation with on-pellet digestion (AP-OPD), in-trap digestion (S-TRAP), and in-solution digestion with SDC (ISD-SDC). (B) Box plots of technical variation per method (CV%). (C) Hierarchical clustering heatmap of relative protein abundances in log_2_ scale. (D) Bar plots showing the number of proteins decreased or enriched by 1.5-fold and 2-fold relative to the average, per method. Error bars show standard deviation. (E) Bar plots showing the number of peptides decreased or enriched by 1.5-fold and 2-fold relative to the average, per method. Error bars show standard deviation. (F) Box plots showing the relative protein abundances (log_2_) for different protein mass ranges, per method. (G) Box plots showing the relative protein abundances (log_2_) at different numbers of transmembrane domain ranges, per method.

To assess the performance of the in-solution SDC digestion method (ISD-SDC), we designed a deep quantitative proteomics experiment using TMTpro-16plex to directly compare this method with various widely used sample preparation methods (Figure 1A). These include in-solution urea digestion (ISD-UREA), acetone protein precipitation with on-pellet digestion (AP-OPD), and acidic methanol protein particulate suspension with on-trap filter digestion (S-TRAP). The urea and acetone precipitation methods were selected as they represent popular approaches in the literature, and the S-TRAP was selected as a representative of the bead-based protein capture approaches and on the basis of previous experience with the protocol. For all comparisons, the different protocols were performed starting from replicate cell pellets of equal numbers of HeLa cells (∼3 million cells), and four replicates were used for each method. Protein aliquots of 100 μg were taken after lysis for downstream processing with the four methods. To determine labeling efficiency and estimate the total peptide amounts recovered after each sample preparation, an initial “TMT label-check” step was performed by premixing small equal aliquots from each sample after the TMT labeling, followed by single-shot LC-MS analysis. Labeling efficiency of >99% was achieved for this 16plex experiment as determined by the percentage of labeled peptides identified using TMTpro as dynamic modification in the database search (Figure S1A). With the same digests, we also performed four separate 4-plex TMT MS2 runs (×2 injections for each method), which showed that all methods had a mean trypsin cleavage efficiency >95% and TMT labeling efficiency >98% (Figure S1B). Although the SDS-based methods initially demonstrated higher total protein recovery (Figure S1C), the ISD-SDC showed the highest peptide recovery after processing, evidenced from the total TMT-signal intensities in the un-normalized samples of the 16-plex prerun (Figure S1D, left panel). A detailed flowchart and indicative timings for each of the different methods is shown in Table S1.

For deep proteome comparison using high pH fractionation and LC-MS analysis, all samples were equalized for peptide amounts based on the total TMT signal per sample (Figure S1D, right panel). A total of 7162 protein groups were quantitated by SPS-MS3 analysis (Table S2). To evaluate the reproducibility and determine technical variation in each method, we visualized the distribution of coefficients of variation (CV%) in box plots using all quantified proteins in the four replicates (Figure 1B). This showed that the ISD-SDC had the lowest technical variability with a median CV = 3.9%.

To assess method-associated bias in protein recovery, we determined the deviation-from-average ratios by calculating sample protein abundance differences from the average of all methods (sample/average, log_2_); a heatmap of these is shown in Figure 1C. Histograms showing the distribution of these values per method are shown in Figure S1E. The interval that represents the percentage of proteins that do not deviate more than 2-fold from the average shows that the ISD-SDC method has the highest percentage of proteins within the interval (97.4%) indicative of the smallest method bias. This was consistent with the number of proteins decreased or enriched by 1.5- or 2-fold across the four methods, with the ISD-SDC method showing the smallest number of decreased proteins or peptides (Figure 1D and 1E).

To investigate the proteins that were decreased or enriched, we evaluated the deviation-from-average ratios by grouping the proteins according to protein mass (size) and the presence of transmembrane domains. The quantitative data revealed a reduced amount of small-sized proteins (<20 kDa) in the S-TRAP method in comparison to in-solution or on-pellet digestion methods (Figure 1F). Furthermore, measurements using the ISD-SDC method showed the best representation of transmembrane proteins compared to other protocols (Figure 1G) consistent with previous studies.^31−33^

Overall, the simplified ISD-SDC sample preparation protocol for isobaric labeling proteomics showed excellent reproducibility with low method-specific protein and peptide bias.

### Development of a Simplified Sample Preparation Workflow for Isobaric Tagging Analysis of 96 Proteomes

**Figure 2:**
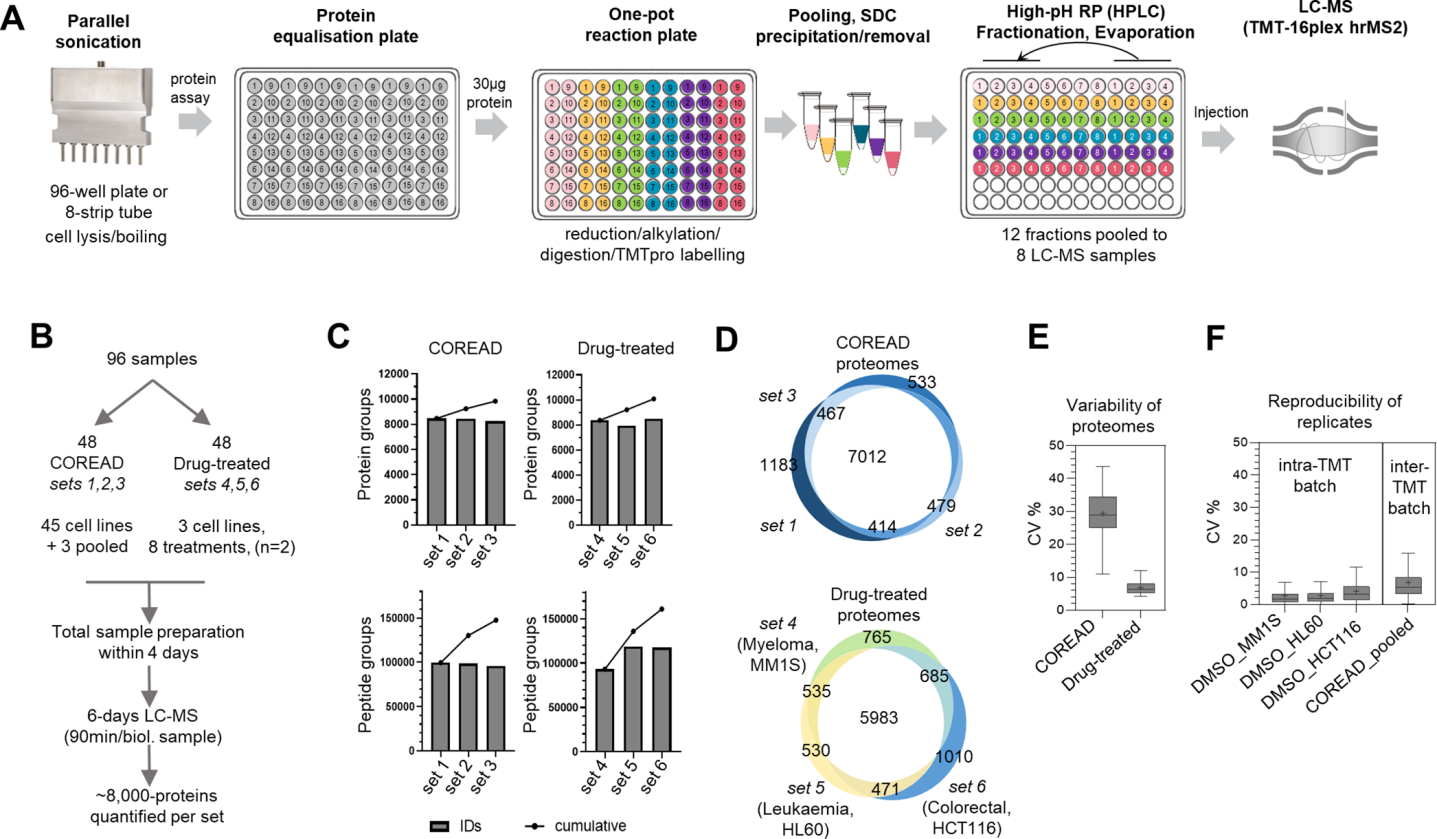
Development of a multibatch isobaric tagging workflow for quantitative proteomics (SimPLIT). (A) Overview of sample preparation workflow for quantitative proteomic analysis of 96 samples from cell pellets. (B) Study design for the analysis of COREAD and drug-treated cell lines using the SimPLIT workflow. (C) Bar plots showing the number of protein groups (top panels) and peptide groups (bottom panels) identified per multiplexed set. (D) Venn diagrams showing the number of proteins identified in three sets per cell line group. (E) Box plots showing the variability of proteomes in the two cell line groups. (F) Box plots summarizing the coefficient of variation of protein abundance for the intra-TMT set (DMSO-treated biological replicates) and inter-TMT sets (pooled COREAD technical replicates).

To increase the sample throughput, we developed a simplified sample preparation workflow for the isobaric tagging analysis of 96 proteomes (SimPLIT) that minimizes sample preparation time by parallel processing. We used the ISD-SDC-TMT protocol, as the one-pot chemistry feature allows the easy implementation of multibatch reactions in simple steps. In addition, we streamlined the offline peptide fractionation to enable fast LC-MS analysis per sample (equivalent to 90 min/sample) while maintaining deep proteome coverage of over 7000 proteins. The entire sample preparation workflow is summarized in Figure 2A.

The fast processing of large numbers of samples is usually hindered by the cell lysis and protein normalization steps that require intensive hands-on time and the handling of multiple sample tubes. To significantly reduce the time required for these steps, we utilize an 8-horn sonication probe that allows simultaneous processing of samples in a standard PCR eight-tube strip or 96-well plate. Overall, the sample solubilization, sonication, and boiling steps require a processing time of about 30 min for 96 samples. Sample heating was included in the workflow to facilitate protein denaturation; however, caution should be taken with higher cell numbers as proteins tend to aggregate at high concentrations and an additional round of sonication may be required for sample homogenization. For the protein normalization steps, we use a direct rapid BCA well-plate assay. With the aid of a multichannel pipet, we deliver equal volumes of samples onto a dilution plate that contains variable volumes of buffer to dilute the samples to the same protein concentration. After mixing, equal volumes of normalized samples that contain 30 μg of proteins are transferred onto a reaction plate, where successive addition of reduction, alkylation, trypsin, and TMT labeling reagents are performed on the same vessel (PCR 8-tube strips or 96-well plates).

Typically, every additional TMT-plex set needs to be fractionated separately on a different well-plate before fractions are transferred and pooled into a smaller number of tubes or vials. Here, to increase throughput and reduce potential peptide losses, all the six TMT-16plex sets are fractionated, evaporated, resuspended, and injected for LC-MS using the same deep well-plate. Twelve fractions are collected per set and pooled into eight by combining the first and last four peptide fractions. For each set, the eight peptide fractions are analyzed using a TMTpro-HRMS^2^ 3 h acquisition method. Overall, 96 proteomes comprising six TMT sets require 3–4 days of total sample preparation including fractionation and 7 days of LC-MS instrument time, which also includes quality control assessments and blank runs.

To rigorously evaluate the workflow on two potential large-scale applications, we prepared 96 samples from cell pellets using the optimized sample preparation method. The study design is shown in Figure 2B. This included proteome profiling of a panel of colorectal cancer cell lines (COREAD 45 cell lines and 3 pooled samples) and a panel of drug-treated cell lines (8 treatments × 3 cell lines × 2 replicates). The COREAD cell lines were previously characterized in our lab using deep TMT10plex-MS3 analysis,^23^ which can be used as a benchmark against the SimPLIT workflow. In addition, published proteomic profiles of IMiDs/CELMoDs also provide comparative benchmark for the drug-treated experiments.^34−41^

We obtained relative quantification for an average of 8324 protein groups and 103 903 peptides across the 96 samples (Figure 2C, Table S3, and Table S4), demonstrating comprehensive proteome coverage. For the COREAD experiments, an average number of 8385 proteins were identified, of which 7012 proteins were quantified without missing values in the 48 cell lines (Figure 2D). In the drug-treated experiments, an average number of 8264 proteins were quantified, with 5983 proteins common in the three different cell lineages (HCT116, MM1S, and HL60) (Figure 2D). The smaller overlap in the drug-treated proteomes is indicative of the tissue of origin specific protein expression across the three distinct cell models.

Overall, the proteomic profiles of the colorectal cancer cell lines displayed much higher biological heterogeneity compared to the less variable drug treatments (Figure 2E). The latter suggests that in cells for drug-treated proteomic profiles, small molecule degraders perturb only a small number of proteins after a short (4 h) drug exposure.

To evaluate the reproducibility of the obtained data from large scale analysis, we determined the median CV% of the protein abundances of samples measured as biological or technical replicates. Within the same TMT set, the median CV was 2.1% measured from the DMSO-treated replicates, while the interbatch median CV of the COREAD pooled sample was 5.3% (Figure 2F). This variation is comparable with the low throughput method (Figure 1B) indicating that preparing large numbers of samples using this high throughput workflow did not affect the reproducibility across TMT batches.

### Benchmarking the SimPLIT Workflow for Large-Scale Quantitation

**Figure 3:**
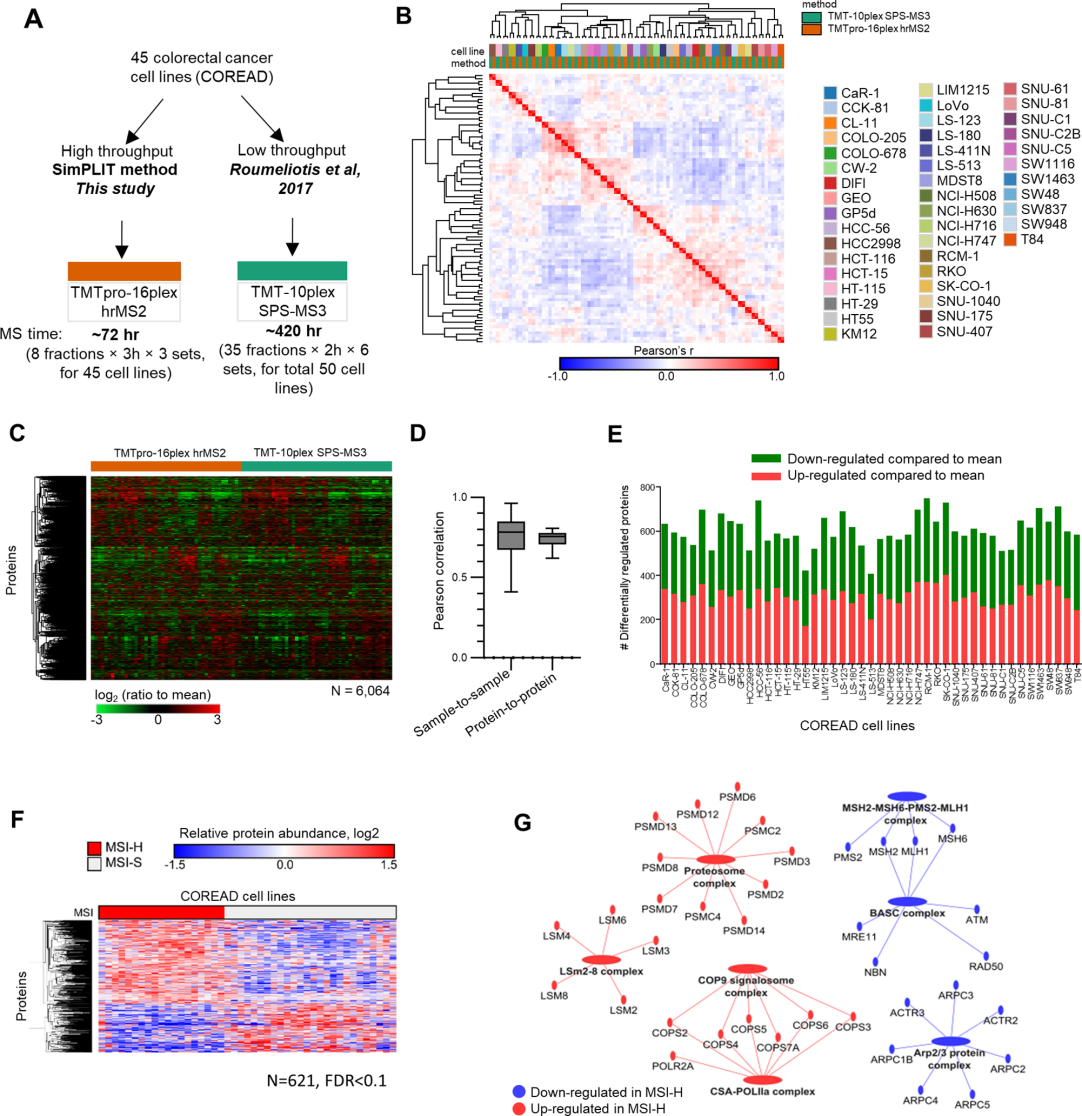
Benchmarking the SimPLIT workflow for large-scale quantitation with colorectal cancer cell lines. (A) Data sets for benchmarking SimPLIT for large scale proteome quantitation. (B) Clustered correlation matrix showing pairing of the COREAD cell lines measured with two different methods (this study and published data). (C) Heatmap of relative protein abundances for 45 colorectal cell lines measured in two different sample preparation and quantitation methods. (D) Boxplots of sample-wise and protein-wise Pearson correlation between interstudy replicates. (E) Bar plots showing the number of differentially regulated proteins per cell line. (F) Heatmap of MSI-high associated proteins (Welch’s two-sided *t* test, FDR < 0.1). Rows represent proteins and columns represent colorectal cancer cell lines. (G) Network showing MSI-high associated proteins and complexes.

To benchmark the performance of SimPLIT for large-scale quantitation, we compared the protein abundance profiles of the 45 cell lines with the previously published TMT-10plex MS3 data (Figure 3A).^23^ Clustered pairwise correlation matrix of 90 proteomes shows pairing of the same cell line from the two different data sets (Figure 3B). This indicates high reproducibility between the studies, despite the differences in sample preparation, quantitation reagents, and multiplexing design. Previously, we used the proteomic profiles of the COREAD models to generate protein correlation networks across the entire panel.^23^ Here, we combined the replicate data sets to assess the proteomic heterogeneity of individual colorectal cancer cell lines. To combine the data sets, we adjusted the log_2_-scaled differences by column *z*-score normalization in order to account for the dynamic range differences between the MS2 and MS3 data (Figure 3C). A total of 6064 quantified proteins were combined from the two data sets without missing values with a median sample-wise or protein-wise Pearson’s correlation of 0.78 and 0.76 respectively (Figure 3D).

Having generated a replicate COREAD proteomics data set, we were interested in identifying the highly dysregulated proteins for each cell line. To determine the cell line-specific signature proteins, we compared the mean abundance of each cell line relative to the mean of 45 cell lines. Figure 3E displays the number of differentially expressed proteins per cell line with the following thresholds: one-sample *t* test *p* < 0.05 and absolute mean relative log_2_ ratio >0.5. The overlap of the differentially regulated proteins across cell lines was small, as we detected only 252 proteins differentially regulated in at least ∼20% of the measured COREAD cell lines; these were predominantly enriched for metabolic processes (Table S5). These also included known tumor suppressors (TP53, MLH1, ERCC5, and CHEK2) and oncogenes (MLLT4, HIP1, RNF213, ZMYM2, CDX2, IDH2, MSI2, HOOK3, GPHN, NFKB2, SEPTIN6) according to MSigDB^42^ protein family annotations (Figure S2A).

Prompted by the frequent differential regulation of MLH1, a key DNA repair protein casually linked to hereditary nonpolyposis colon cancer and microsatellite instability (MSI), we further explored the proteomic signatures of cell lines that were either MSI-high or microsatellite stable (MSS) according to previously assembled annotation.^43^ This genetic marker is used for colorectal tumor classification as well as for making treatment decisions with T cell checkpoint inhibitors such as pembrolizumab.^44^ High MSI is attributed to a defective DNA mismatch repair system that induces frequent mutations proximal to short repetitive DNA microsatellite sequences.^45^ We identified 621 differentially expressed proteins between the MSI-H and MSS (microsatellite stable, MSI-low) cell lines (Welch’s two-sided *t* test; permutation-based FDR < 0.1; Figure 3F, Table S6). The MSI-high associated proteins ranked according to MSI-H/MSS ratio (log_2_) from each direction (top 20) are shown in Figure S2B and include the key apoptotic proteins BAX and PYCARD that were found in lower abundance in MSI-H cells. Overall, the proteomic profiles of MSI-H cells were consistent with deficient DNA mismatch repair and upregulation of RNA processing and protein degradation (Figure 3G) as previously described.^9,23^ Further, we show an upregulation of protein complex components of the NEDD8-activating enzyme (NAE1 and UBA3), deneddylation (COP9 signalosome complexes), sumoylation (SAE1 and UBA2), and proteasomal systems. This suggests an overall upregulation of the ubiquitin-proteasome dependent protein turnover machinery in MSI-H cells. Recent work by McGrail et al. show a high dependency of MSI cancers on protein clearance systems and implicate this axis as a therapeutic vulnerability in MSI cancers.^46^

Taken together our analysis shows that the SimPLIT workflow offers a time-efficient approach to capture proteomes that are comparable to measurements made with well-established quantitative deep proteomics methods.

### High Throughput Degradation Profiling of Ubiquitin Ligase Modifying Compounds Using SimPLIT

Small molecules inducing protein degradation by ubiquitin ligase substrate modulation bear promising opportunities for previously intractable targets.^47^ A key bottleneck in the development of these small molecule protein degraders (e.g., molecular glues and proteolysis targeting chimeras, PROTACs) is the availability of rapid and cost-effective proteomic assays to identify the drug-induced neosubstrates. The SimPLIT proteomics workflow is well suited to address this application, offering scalability and fast data acquisition to generate highly reproducible deep proteomic profiles of degrader compounds.

**Figure 4:**
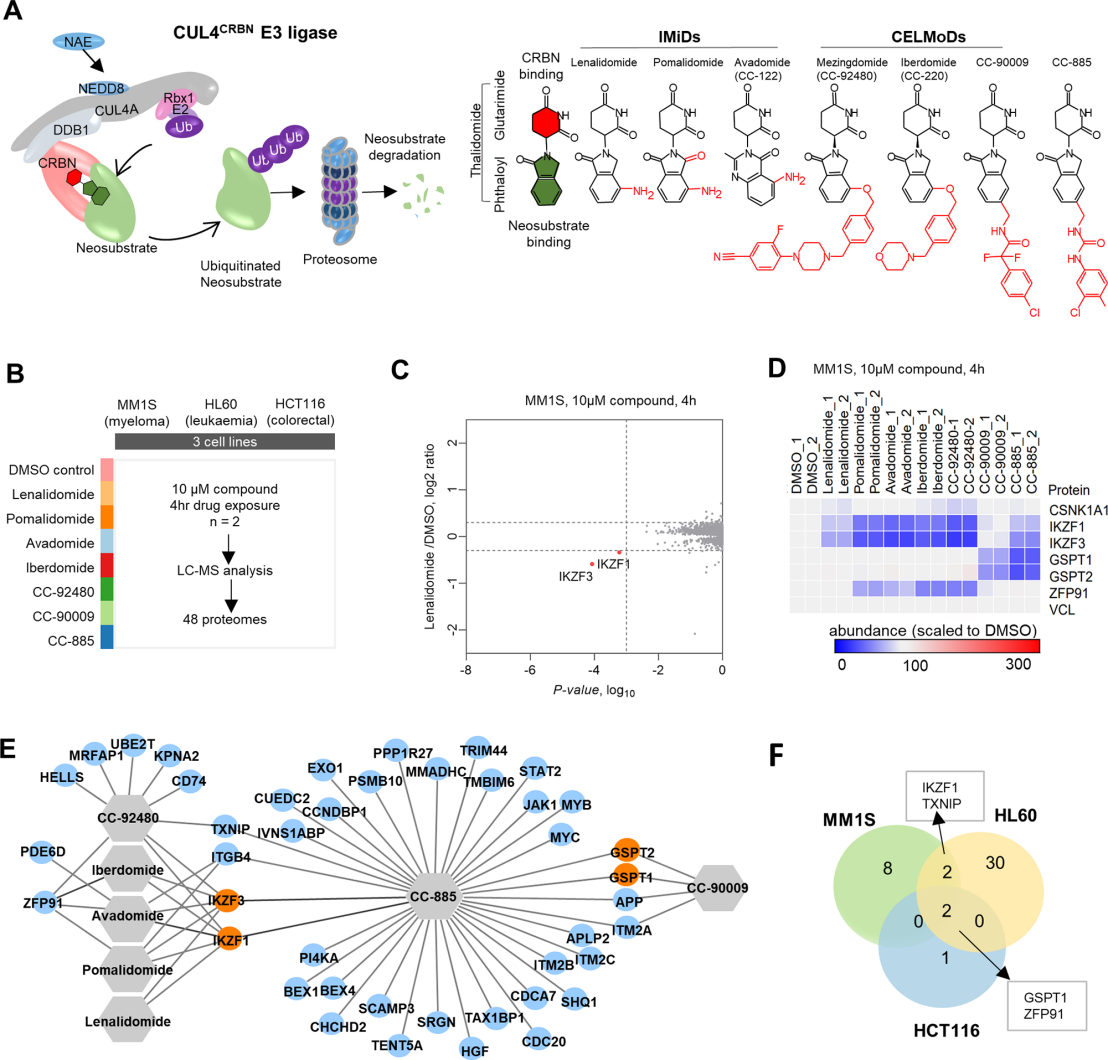
Target degradation landscape of clinical cereblon modulators. (A) A graphical model of IMiDs/CELMoDs mechanism of action and their chemical structures. Glutarimide moiety (red) binds to CRBN, the substrate receptor of E3 ligase, to recruit neosubstrates for ubiquitination and proteasomal degradation. Chemical substitutions of thalidomide lead to changes in neosubstrate degradation profiles. (B) Experimental design for proteomic profiling of molecular degraders in three cell lines. (C) Representative volcano plot highlighting lenalidomide-dependent degradation targets in MM1S. (D) Representative heatmap showing relative protein levels in MM1S after a 4 h exposure to IMiD/CelMOD. Known neosubstrates and vinculin loading control are shown. (E) Protein-drug network showing common and unique degraded targets between IMiDs/CELMoDs in three cell lines. Known neosubstrates are illustrated as orange nodes. (F) Venn diagram displaying the overlap of drug-induced degradation targets between cell lines.

As a proof of concept, we leveraged the large sample capacity of the SimPLIT platform to perform global proteomic screens to identify degrader targets in three cell lines from different tissue origins: multiple myeloma (MM1S), leukemia (HL60), and colorectal cancer (HCT116). We evaluated the degradation profiles of seven cereblon E3 ligase (CRL4^CRBN^) modulators (IMiDs/CELMoDs) (Figure 4A).^34,35,38,39,48^ These compounds, also referred to as “molecular glue degraders”, bind to cereblon, the substrate receptor of the CUL4^CRBN^ E3 ligase, and redirect its substrate specificity to induce the binding, ubiquitination, and subsequent proteasomal degradation of neosubstrates (Figure 4A).^37,49−51^

The three cell lines were treated with 10 μM of lenalidomide, pomalidomide, avadomide, iberdomide, CC-92480, CC-90009, CC-885, or DMSO control all in replicates (Figure 4B). The proteomes were analyzed after 4 h of treatment to detect primary degraded targets and minimize the occurrence of secondary events. In order to identify potential degradation targets/neosubstrates, we measured the change in protein abundance of the drug-treated cells relative to DMSO controls. To report differentially regulated proteins, the data were filtered to an abundance decrease by at least 25% relative to DMSO (log_2_FC < −0.32, *p*-value <0.001 using *t* test, PD 2.4) and identified with at least two unique peptides. The volcano plots highlighting the differentially regulated proteins for each drug-cell line treatment are shown in Figures S3A, S4A, S5A. Overall, the majority of these molecular degraders are highly selective on the basis of the number of downregulated proteins. In lenalidomide-treated MM1S cells, only 2 out of the 7969 quantitated proteins were significantly perturbed (Figure 4C). Both hits (IKZF1 and IKZF3) are established therapeutic targets of lenalidomide in multiple myeloma.^37^ The proteomic profiles generated across the seven molecules in MM1S (Figure 4D) show drug-dependent degradation of the known neosubstrates including: transcription factors Ikaros (IKZF1) and Aiolos, (IKZF3), E3 ubiquitin-protein ligase ZFP91, and the translation termination proteins GSPT1 and GSPT2 (with vinculin, VCL, as loading control).^37,39,52^

We recovered 43 proteins that were downregulated across the 7 molecules in 3 cell lines (Figure 4E and Figure S6). In addition to the known neosubstrates, the most frequently down-regulated proteins included ITGB4, APP, TXNIP, and ITM2A. In normal conditions, APP is an endogenous degradation substrate of CRBN.^53,54^ Additionally, down-regulated proteins of this panel of compounds include oncogenes (CD74, IKZF1, MYC, MYB, and JAK1), transcription factors (STAT2, ZFP91, IKZF1, IKZF3, MYC, MYB), cell differentiation markers (ITGB4 and CD74), and the HGF growth factor (Figure S6). In all the three cells, GSPT1 and ZFP91 were degraded consistently (Figure 4F).

### Structure–Target Degradation Relationships of Cereblon E3 Ligase Modulators

**Figure 5:**
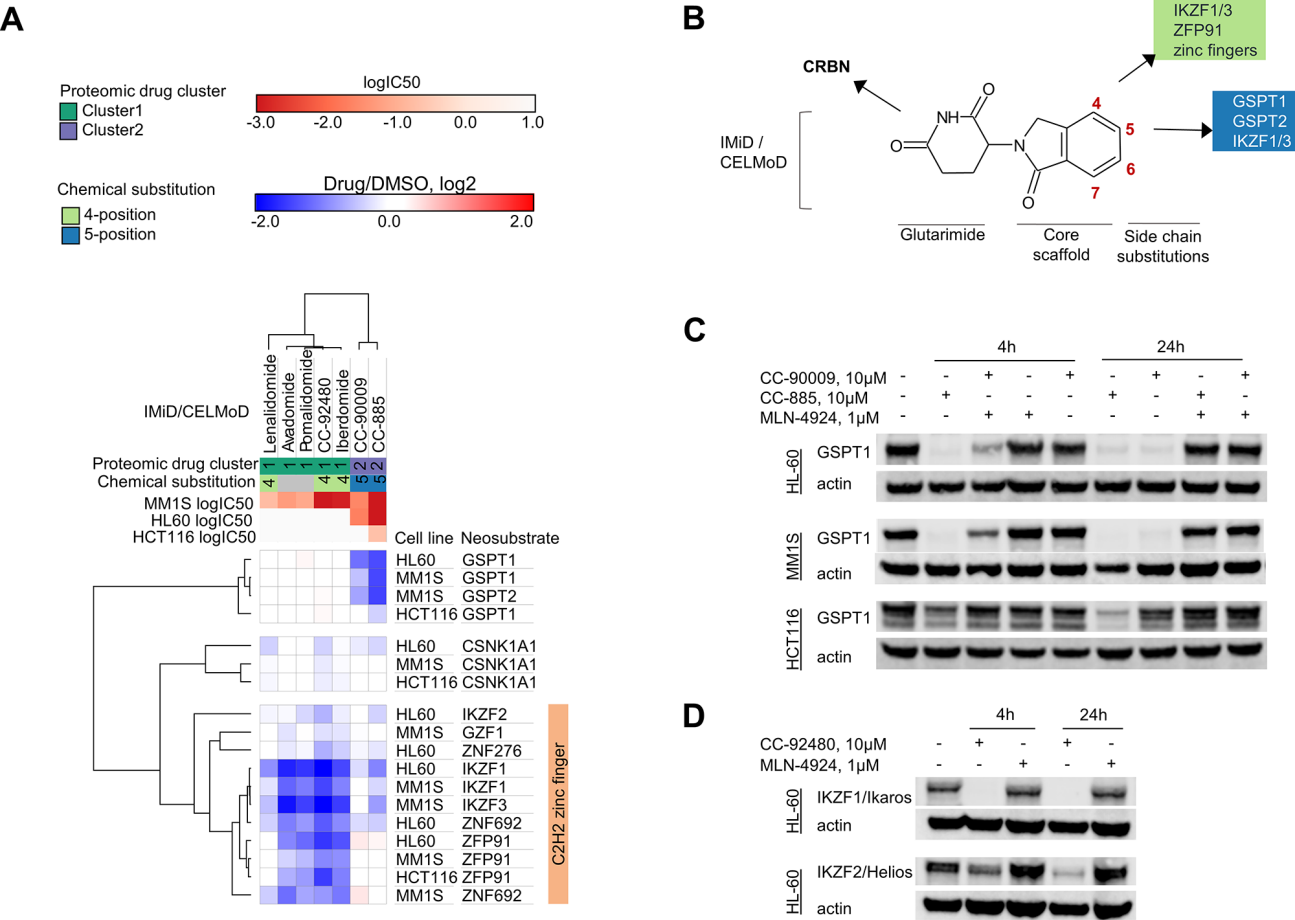
Structure, target, and activity relationships of cereblon-binding compounds. (A) Clustered heatmap of the protein abundance of known targets after drug treatment in three cell lines aligned with color-coded drug clusters, chemical substitutions, and cellular activities (log IC50). (B) Summary of relationships between chemical substitution position and target engagement/degradation for the IMiDs/CELMoDs used in the study. (C) Western blot validation of GSPT1 profile in the three cell lines upon treatment with CC-90009, CC-885, and MLN-4924. (D) Western blot validation of IKZF1 and IKZF2 profiles in HL60 cells upon treatment with CC-92480 and MLN-4924.

CRBN hijacking molecules have a very complex structure/target degradation relationship, as small structural modifications induce changes in the profile of degraded substrates.^55^ Large scale deep protein profiling offers the opportunity to interrogate relationships between molecular degraders and their targets to deconvolute their structural selectivity attributes. As a proof of concept, to show that our SimPLIT method would enable these structure/target degradation explorations, we mined the data from our small panel of thalidomide analogues using their known degradation targets.^34,37,39,52,56−58^ Clustering of the protein abundance changes (drug/DMSO, log_2_) in three cell lines separated the compounds into two proteomic drug clusters: cluster1 (lenalidomide, pomalidomide, avadomide, iberdomide, CC-92480) that degrades C2H2 zinc finger proteins, and cluster2 (CC-90009 and CC-885) that degrades translation termination proteins GSPT1 and GSPT2 (Figure 5A). Moreover, the chemical substitution of these analogues in either position 4 or 5 of the common phthalimide or isoindolinone substructures overlap with proteomic cluster1 and cluster2, respectively (Figure 5A). In agreement with previous studies, the proteomic clusters show that minor structural changes of CRBN binders can selectively alter the substrate specificity (Figure 5B).^40^

In parallel to the target degradation screen, we also performed a phenotypic viability screen using the same group of compounds and cell lines (experimental design in Figure S7A). To explore the target and cellular activity relationships, we annotated the clustered heatmaps with cellular activity (log IC50) (Figure 5A) using a five-day CellTiter-Blue cell viability assay (dose–response curves in Figures S3B, S4B, S5B). We considered the compound as active (shown as red) when the IC_50_ is less than 10 μM and it inhibits cell proliferation by at least 50%. The active compounds in cluster1 match with IKZF1 and IKZF3 degradation in MM1S (Figure 5A, Figure S3, and Figure S4).^34−38^ In addition, the active compounds in cluster2 match with GSPT1 degradation in all cell lines (Figure 5A, Figure S5). Both CC-90009 and CC-885 degrade GSPT1 and inhibit proliferation in MM1S and HL60 as expected (Figure S5).^39−41^ Notably, in both HL60 and MM1S, the CC-885 treatments show a higher number of degradation targets compared to CC-90009, exposing the different selectivities of these GSPT1 degraders (Figure S5). The in vivo toxicity reported for CC-885^59^ may be associated with these observed off-target effects and would support a preference of the more selective GSPT1 degrader (CC-90009) in clinical trials for acute myeloid leukemia (AML)^41,59^ (in clinical trials #NCT02848001, #NCT04336982).

Our data show that drug-induced protein degradation events can be variable across different cell lines. For example, CC-90009 rapidly degrades GSPT1 in MM1S and HL60 but does not degrade it in HCT116. GSPT1 degradation in HCT116 was not observed at a longer time point (24 h) by immunoblotting (Figure 5C). This indicates that neosubstrate expression is not in itself a reliable predictor of degradation outcome in cells and tissues. We also show that the weak degradation of GSPT1 by CC-885 correlates with a modest cell growth inhibition in HCT116 (IC50 = 0.198 μM) (Figure 5C, Figure S5), highlighting that more potent GSPT degraders could be used in colorectal cancer.^60,61^

Some compounds that were shown to be inactive based on phenotypic screens (Figure S7B) may still induce drug-dependent protein degradation events in cells (Figure S7C, S7D). As an example, cluster1 compounds (e.g., CC-92480) do not inhibit HL60 proliferation despite showing strong degradation of IKZF1 (Figure S7E). Interestingly, the proteomic profiles of the transcription factor Helios (IKZF2) in HL60 showed weak degradation with CC-92480 (Figure 5A). A more pronounced degradation of IKZF2 was observed at a longer time point (24 h) (immunoblot, Figure 5D). This IKZF2 degradation is blocked after pretreatment of MLN4924, a neddylation inhibitor of the cullin scaffold in E3 ligases indicating that IKZF2 is a CC-92480 dependent substrate of CUL4^CRBN^ ligase. Recent studies have shown that IKZF2 degraders (ALV2 and DKY709) can modulate regulatory T-cells activity^58^ (clinical trial #NCT03891953). This indicates that compounds with no activity in phenotypic viability screens may provide novel insights when included in degrader target screening in different cell contexts. This shows that global proteomic screening of molecular degraders in different cell lines can be valuable in expanding the association of new targets and broaden therapeutic application even with existing compounds.

### Evaluation of Potential Further Improvements in the SimPLIT Workflow

Lastly, we explored the feasibility of potential further improvements in our workflow, streamlining the processing to reduce the sample preparation time and steps, for potential easy adaptation on an automation platform. These include simultaneous lysis, reduction, and alkylation at a single step by adding TCEP and iodoacetamide in the lysis buffer, addition of a universal nuclease to circumvent the need for strong probe sonication, no use of boiling, and high-pH fractionation with an offline reversed-phase column of smaller dimensions (1.0 × 100 mm) for collection of fractions with smaller volumes and faster drying. To this end, we designed a workflow based on the above modifications of the SimPLIT workflow (in tubes) and analyzed 16 COREAD cell pellets (8 cell lines × 2) in a TMTpro-16plex experiment as described in the detailed flowchart in Figure S8A. For this pilot experiment, we analyzed 6 pooled fractions and quantified 5409 proteins (Table S7) with an HRMS^2^ method (equivalent to 45 min per biological sample). In this experiment, trypsin cleavage efficiency was 96.7% (Figure S8B), TMTpro labeling efficiency was 99.9% (Figure S8C), and carbamidomethylation efficiency of cysteine containing peptides was 98.8% (Figure S8D), with the percentage of peptides with carbamidomethylation side reactions at only 0.22% (Figure S8E). Overall, the efficiencies of the modified SimPLIT workflow are in line with those previously demonstrated. Notably, the median protein CV% between the replicate cell pellets was 1.7% (Figure S8F), with single PSM proteins having a low median CV of 3.7%. These demonstrate excellent and improved reproducibility over the entire range of protein abundances. A heatmap of all quantified proteins is shown in (Figure S8G) and illustrates that the quantification profiles of the two replicate cell pellets per cell line are nearly identical. These preliminary data show that our SimPLIT workflow is amenable to further significant improvements in processing time and reproducibility. All the changes facilitate the adaptation of the workflow to basic liquid handling workstations, which offer a seamless route to near complete automation.

## Discussion

In this study, we have developed and benchmarked a simplified isobaric tagging workflow for large-scale multibatch quantitative proteomic analysis of cell lines. Our method uses the same SDC-based buffer from cell lysis to isobaric labeling, thereby enabling successive addition of reagents onto a single vessel in the smallest possible number of steps. The final single detergent removal step by acid precipitation is implemented after combining the TMT-labeled peptides. This strategy minimizes sample-to-sample processing variation between protein assay and peptide fractionation steps. Moreover, cost from filters, beads or peptide cleanup resins is reduced, as no individual sample cleanup is performed. We demonstrate that our protocol has excellent proteome representation, high reproducibility, and low method-specific protein bias when compared to widely used alternative workflows. In particular, low molecular weight and transmembrane proteins are reliably measured by our simplified in-solution digestion method.

The simplified TMT preparation method allows easy and cost-effective implementation in a 96-well format or strip PCR tube array in any proteomics laboratory where liquid-handling platforms are not available. If such platforms are available, our workflow can be easily adapted for large-scale automated processing from cell lysis to TMT labeling. We provide preliminary data from further optimizations demonstrating a decrease in sample preparation time and significantly improved reproducibility. These include (a) one-step simultaneous reduction and alkylation by adding TCEP and iodoacetamide in the SDC-based lysis buffer followed by 45 min incubation at room temperature, (b) addition of a universal nuclease that can reduce the sonication time, and (c) use of reversed-phase columns with smaller dimensions (e.g., 1.0 × 100 mm) for off-line fractionation and operation at lower flow rates that will result in smaller fraction volumes and faster drying. Further reduction in the MS analysis time could be achieved using high-throughput LC systems with reduced injection cycle overheads.^62^ Additionally, given the lossless processing steps of the workflow, a smaller number of cells can be analyzed, extending the applicability. In the current SimPLIT manual handling approach, protein equalization after protein assay remains the only time-consuming step in sample preparation. On the basis of the work presented here and the identified rate limiting steps, we envision a semiautomated SimPLIT platform with the following main steps prior to fractionation and LC-MS analysis: (1) one-step cell lysis/reduction/alkylation in a 96-well-plate, (2) protein assay, (3) protein concentration equalization, aliquoting, and trypsin digestion using an affordable liquid handling system to deliver variable volumes, and (4) TMT labeling/pooling/SDC removal, where steps 1, 2, and 4 can be easily performed with a multichannel pipet.

Given that our workflow has the capabilities to expedite proteomic data acquisition, medium- to larger-scale applications in biomarker and drug target discovery can be made easier with fewer resources. We showcased this by investigating proteomic heterogeneity of a panel of colorectal cancer cell lines and by performing target discovery for a set of molecular degraders in different cell lines.

In addition to identifying novel targets, the advantage of large-scale MS-based proteomic profiling over traditional Western blotting of specific targets is that it allows accurate quantitative comparison of multiple active compounds and unknown regulated targets. This can reveal off-target effects and prioritize more selective compounds on the basis of the number of degraded or indirectly regulated proteins. We also show that this approach allows the interrogation of the chemical structure and activity relationships on the basis of proteome-wide perturbations. For example, we show that selectivity of target degradation changes as the position of the chemical substitution of thalidomide analogues is varied. We envision the use of proteomic screening platforms for systematic target deconvolution of large chemical libraries with potential degraders and ultimately to guide the rational design of molecular glues, PROTACs, and other compounds targeting the UPS systems.

Our data demonstrate that drug-induced protein degradation events are not consistent across different cell lineages. This indicates that neosubstrate expression or binding to the drug-CRBN complex do not always predict cellular degradation. Cell-dependent differences in target expression,^63,64^ competition with other substrates,^65^ and the availability of the E3 ligase and ubiquitin-proteasome system machinery contribute to influence target degradation events.^66−68^ As proteomics screening of compounds in a wide panel of cell lines is expensive, a more focused panel of cell lines with distinct proteomic features can be used (e.g., COREAD). Further, as a large number of inactive compounds from phenotypic viability screens also induce drug-dependent degradation, compounds with no cellular activity in these assays (e.g., CC-92480 in HL60) should not necessarily be excluded for degradation target screens.

In summary, we provide a reliable, less laborious, and more cost-effective workflow that can be easily adopted by any proteomics lab for medium-to-large scale TMT-based studies involving the analysis of cell lines.

## Supporting Information Available

Detailed description of the multiplexed comparison of the four different methods (Supplementary Methods), Supplementary Figures S1–S8 and Western blotting analysis; Figure S1: Qualitative and quantitative characteristics of methods comparisons; Figure S2: Dysregulated and MSI-high associated proteins in colorectal cancer cell lines; Figure S3: Proteomic profiles and cellular activities of lenalidomide and pomalidomide; Figure S4: Proteomic profiles and cellular activities of avadomide, iberdomide, and CC-92480; Figure S5: Proteomic profiles and cellular activities of CC-90009 and CC-885; Figure S6: Protein degradation profiles of IMiDs/CELMoDs in three different cell lines; Figure S7: Summary of degradation targets and drug activity; Figure S8: Evaluation of further improvements in the SimPLIT workflow (PDF)
Table S1: Detailed flowcharts for the different methods and indicative timings; Table S2: Protein abundance data from the method comparison experiment measured by TMTpro-SPS-MS3; Table S3: Protein abundance data for 45 COREAD cell lines and three pooled controls measured by TMTpro-HRMS^2^ using the SimPLIT method; Table S4: Protein abundance data for drug-treated cell lines (MM1S, HL60, HCT116) measured by TMTpro-HRMS^2^ using the SimPLIT method; Table S5: Differentially regulated proteins in at least ∼20% of the measured COREAD cell lines and GO-BP enrichment analysis by STRING;^69^ Table S6: MSI-high associated proteins in colorectal cancer cell lines; Table S7: Protein abundance data from the in-lysis reduction/alkylation TMTpro16plex experiment (ZIP)

## Notes

The mass spectrometry proteomics data have been deposited to the ProteomeXchange Consortium via the PRIDE^70^ partner repository with the data set identifiers PXD031510 and PXD034022.
