# Supplementary material for: SimPLIT: Simplified Sample Preparation for Large-Scale Isobaric Tagging Proteomics
Source: J Proteome Res. 2022 Jul 18;21(8):1842–56. doi: 10.1021/acs.jproteome.2c00092 (PMC9361352; doi:10.1021/acs.jproteome.2c00092)
Supplement: Supplementary file 1 — pr2c00092_si_001.pdf [file pr2c00092_si_001.pdf]

Supporting Information

SimPLIT: Simplified sample preparation for large-scale isobaric tagging proteomics

Fernando J. Sialana[1,2], Theodoros I. Roumeliotis[1,*], Habib Bouguenina[2], Laura Chan Wah Hak[2], Hannah Wang[2], John Caldwell[2], Ian Collins[2], Rajesh Chopra[2], Jyoti S. Choudhary[1,*]

[1]Functional Proteomics Group, The Institute of Cancer Research, Chester Beatty Laboratories, London SW3 6JB, UK.

[2]Cancer Research UK Cancer Therapeutics Unit, The Institute of Cancer Research, London SM2 5NG, UK.

[*]Correspondence should be addressed to J.S.C (jyoti.choudhary@icr.ac.uk) and T.I.R (theo.roumeliotis@icr.ac.uk)

Contents

Supplementary methods 2

Supplementary figures 4

Western blotting 11

Supplementary table captions 14

Supplementary methods

Methods comparison: sample preparation for proteomic analysis and LC-MS analysis

Cell homogenization, protein assay and alkylation. HeLa cell pellets of four million cells each, were homogenized with 150 µL lysis buffers supplemented with protease inhibitors (Thermo, Halt™). The composition of lysis buffers was as follows: a) ISD-UREA: 8M urea,100 mM triethylammonium bicarbonate (TEAB); b) AP-OPD: 1% SDS, 100 mM TEAB; c) S-TRAP: 5% SDS,100 mM TEAB; d) ISD-SDC: 1% sodium deoxycholate (SDC), 100 mM TEAB, 10% isopropanol and 50 mM NaCl. Homogenisation was carried out with probe sonication for 3 × 5 s with pulses of 1 s at 40% amplitude (EpiShear) followed by heating at 90 °C for 5 min except for urea-lysed samples (room temperature). All samples were sonicated again for 5 sec after boiling. Protein concentration was measured with the rapid gold BCA Protein Assay (Pierce) according to the manufacturer's instructions. Aliquots containing 100 µg of total protein were used for protein digestion. The cysteines were reduced with 5 mM tris-2-carboxyethyl phosphine (TCEP) for 1 h at 60°C except for urea-lysed samples (room temperature). The cysteines were blocked by 10 mM iodoacetamide (IAA) for 30 min at room temperature in the dark for all samples.

Acetone precipitation followed by on-pellet digestion (AP-OPD). The on-pellet digestion was carried out according to a manufacturer's protocol for preparing and labelling peptides for TMT isobaric mass tags (Thermo Fisher, USA). One hundred micrograms of the alkylated protein lysates were precipitated by adding six volumes of pre-chilled acetone and precipitated overnight. Samples were centrifuged at 8000 × g for 10 minutes at 4°C. Acetone was discarded and the pellets were allowed to dry for 2 to 3 minutes. The pellets were resuspended in 100 µL of 100 mM TEAB. Three micrograms of trypsin were added per 100 µg of protein and the samples were digested overnight at 37°C with constant shaking at 600 rpm.

In-solution digestion with urea (ISD-UREA). The one hundred micrograms of the alkylated proteins were diluted with 100 mM TEAB to reduce the concentration of urea to 1.6 M. Trypsin aliquots were added (3 µg per 100 µg protein) and digested overnight at 37 °C. The digested samples were acidified to 0.4% TFA, spun for 10 min at 2500 × *g* , and desalted on Pierce C18 Spin Desalting Columns (89852). Peptides were eluted with 50% ACN/0.1% TFA and SpeedVac dried.

In-trap digestion (S-TRAP). Sample preparation of S-Trap micro spin column (ProtiFi, Huntington, NY, USA) was according to the manufacturer's protocol. Fifty micrograms of the alkylated samples in replicates were used per micro spin column. Colloidal protein particles were formed by the addition of phosphoric acid (1.2% final concentration) and S-Trap buffer (90% methanol in 100 mM TEAB, pH 7.1). The mixture was transferred onto the S-Trap microcolumn and centrifuged at 4000 × *g* for

10 min. The samples were washed with a 150 µL S-Trap buffer. Finally, a 25 µL trypsin solution (2.5 µg trypsin in 50 mM TEAB) was added and samples were digested at 37°C overnight. Peptides were eluted with the addition of 40 µL TEAB unto the micro spin columns and centrifuged at 4000 × *g* for 10 min and washed twice with 50 µL 50% ACN containing 0.2% formic acid solution. The digested peptides were SpeedVac dried.

In-solution digestion with sodium deoxycholate (ISD-SDC). One hundred micrograms of alkylated proteins were incubated overnight (37°C) with the addition of 6 µL of trypsin stock (3 µg trypsin) and constant shaking at 600 rpm.

Isobaric labelling of peptides. The resultant peptides were diluted with 100 mM TEAB buffer. Thirty percent of the digested samples were labelled with a tandem mass tag (TMTpro-16plex) reagent (Thermo Scientific) according to the manufacturer instructions. For the SDC-digested samples, the TMT peptide mixture was acidified with 1% formic acid and the precipitated SDC was removed by centrifugation. Finally, all TMT labelled samples were combined in equal amounts to a single tube in two steps. First, to check labelling efficiency, 1 µL of each sample were pooled, desalted and analyzed by LC-MS. After the labelling efficiency and equal loading check, all TMT-labelled samples were pooled at equal peptide amounts and dried with a centrifugal vacuum concentrator.

High pH Reversed-Phase peptide fractionation. Offline peptide fractionation was based on high pH Reverse Phase (RP) chromatography using the Waters XBridge C18 column (2.1 × 150 mm, 3.5 µm) on a Dionex UltiMate 3000 HPLC system at a 0.85% gradient with a flow rate of 0.2 mL/min. Mobile phase A was 0.1% (v/v) ammonium hydroxide, and mobile phase B was 100% acetonitrile, 0.1% (v/v) ammonium hydroxide. Retention time-based fractions were collected and pooled into twenty-eight samples for LC-MS analysis.

LC-MS analysis. LC-MS analysis was performed on the Dionex UltiMate 3000 UHPLC system coupled with the Orbitrap Lumos mass spectrometer (Thermo Scientific). Samples were analyzed with the EASY-Spray C18 capillary column (75 µm × 50 cm, 2 µm) at 50 °C. Mobile phase A was 0.1% formic acid and mobile phase B was 80% acetonitrile, 0.1% formic acid. The gradient separation method was as follows: 90 min gradient from 5% to 38% B, for 10 min up to 95% B, for 10 min isocratic at 95% B, re-equilibration to 5% B in 5 min, for 10 min isocratic at 5% B. Precursors between 375 and 1,500 m/z were selected, with a mass resolution of 120,000, automatic gain control (AGC) of 4×10^5, and IT (injection time) of 50 ms, with the top speed mode in 3 s, and the precursors were isolated for collision-induced dissociation (CID) fragmentation with a quadrupole isolation width of 0.7 Th (Thomson unit). The collision energy was set at 35%, with AGC at 1×10^4 and IT at 50 ms. MS^3 quantification was obtained with higher-energy collisional dissociation (HCD) fragmentation of the top 5 most abundant CID fragments isolated with synchronous precursor selection (SPS). Quadrupole isolation width was set at 0.7

Th, collision energy was applied at 65%, and the AGC setting was 1×10^5 with IT at 105 ms. The HCD MS^3 spectra were acquired for the mass range 100 to 500 with a resolution of 50,000. Targeted precursors were dynamically excluded for further isolation and activation for 45 s with 7 ppm mass tolerance. For single-shot LC-MS analysis, the precursors were isolated for higher-energy C-trap dissociation (HCD) fragmentation with a quadrupole isolation width of 0.7 Th (Thomson unit). The collision energy was set at 34%, with AGC at 1×10^5 and IT at 54 ms. The HCD MS2 spectra for identification and quantification were acquired with a resolution of 30,000. Targeted precursors were dynamically excluded for further isolation and activation for 30 s with 7 ppm mass tolerance.

Supplementary figures

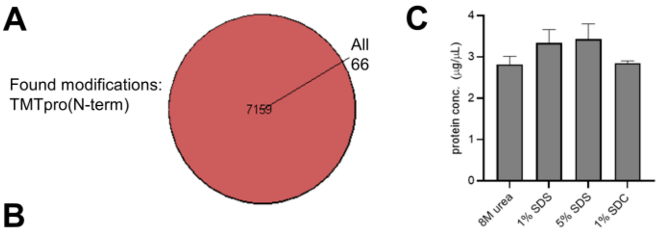

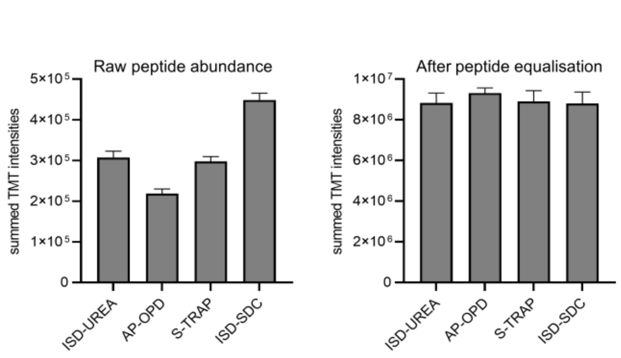

B

	ISD-UREA		AP-OP		S-TRAP		ISD-SDC	
Run#	1	2	1	2	1	2	1	2
Protein groups	1804	1878	1582	1428	1848	1798	1889	1807
Peptide groups	7014	7351	5738	5125	7211	6767	6911	6667
Cleavage efficiency (%)	94.92	95.16	97.51	97.68	98.09	98.12	97.18	97.44
TMT labelling efficiency (%)	99.9	99.9	98.0	98.0	99.9	99.9	99.7	99.9

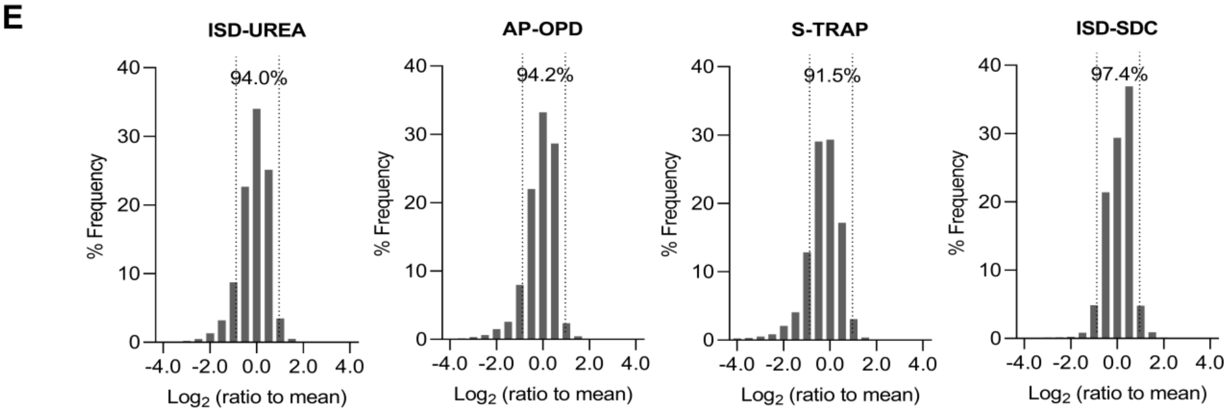

Figure S1. Qualitative and quantitative characteristics of methods comparisons. A) Venn diagram showing the overlap of all peptides and TMTpro-labelled peptides in the TMT-16plex pre-run analysis. B) Summary table showing number of identifications, trypsin digestion and TMT labelling efficiencies for the four 4plex runs of the different methods. C) Bar plots of the protein concentrations of the HeLa cell lysates for the four different lysis methods based on rapid gold BCA Protein Assay (Pierce). Error bars represent standard deviation from 4 replicates. D) Bar plots

highlighting method-specific differences of peptide recoveries analysed by TMT-MS3 before and after peptide equalization without data normalization. Error bars show standard deviation. E) Histograms of relative protein abundances (log2) per method. The dashed lines represent a 2-fold deviation from the average and the percentage (%) of proteins within this interval are indicated.

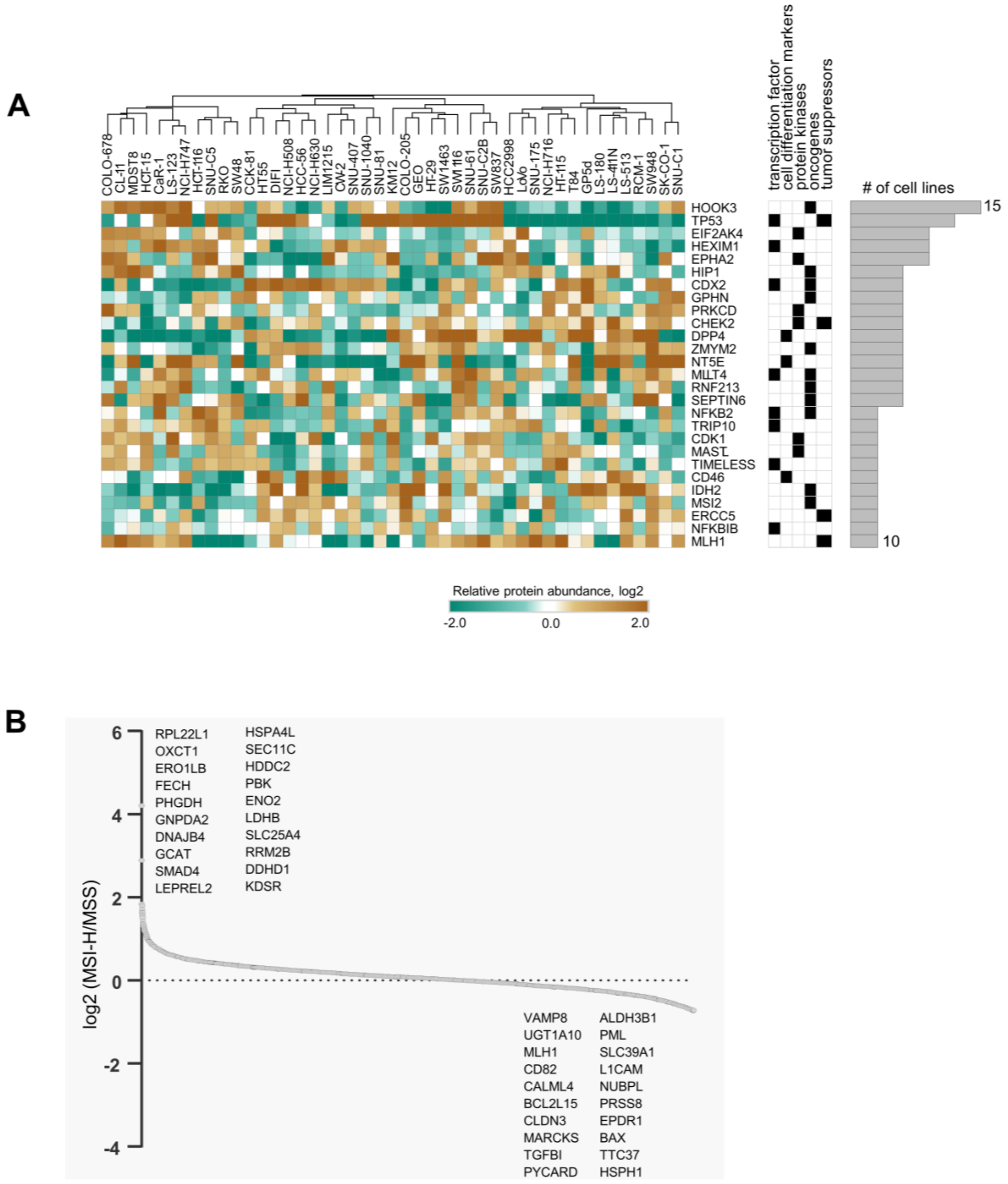

Figure S2. Dysregulated and MSI-high associated proteins in colorectal cancer cell lines. A) Heatmap of frequently dysregulated cancer-related proteins (>20% of colorectal cancer cell lines with protein family annotations from MSigDB). B) MSI-

high associated proteins ranked according to MSI-H/MSS ratio (log2). The top 20 regulated proteins from each direction are listed.

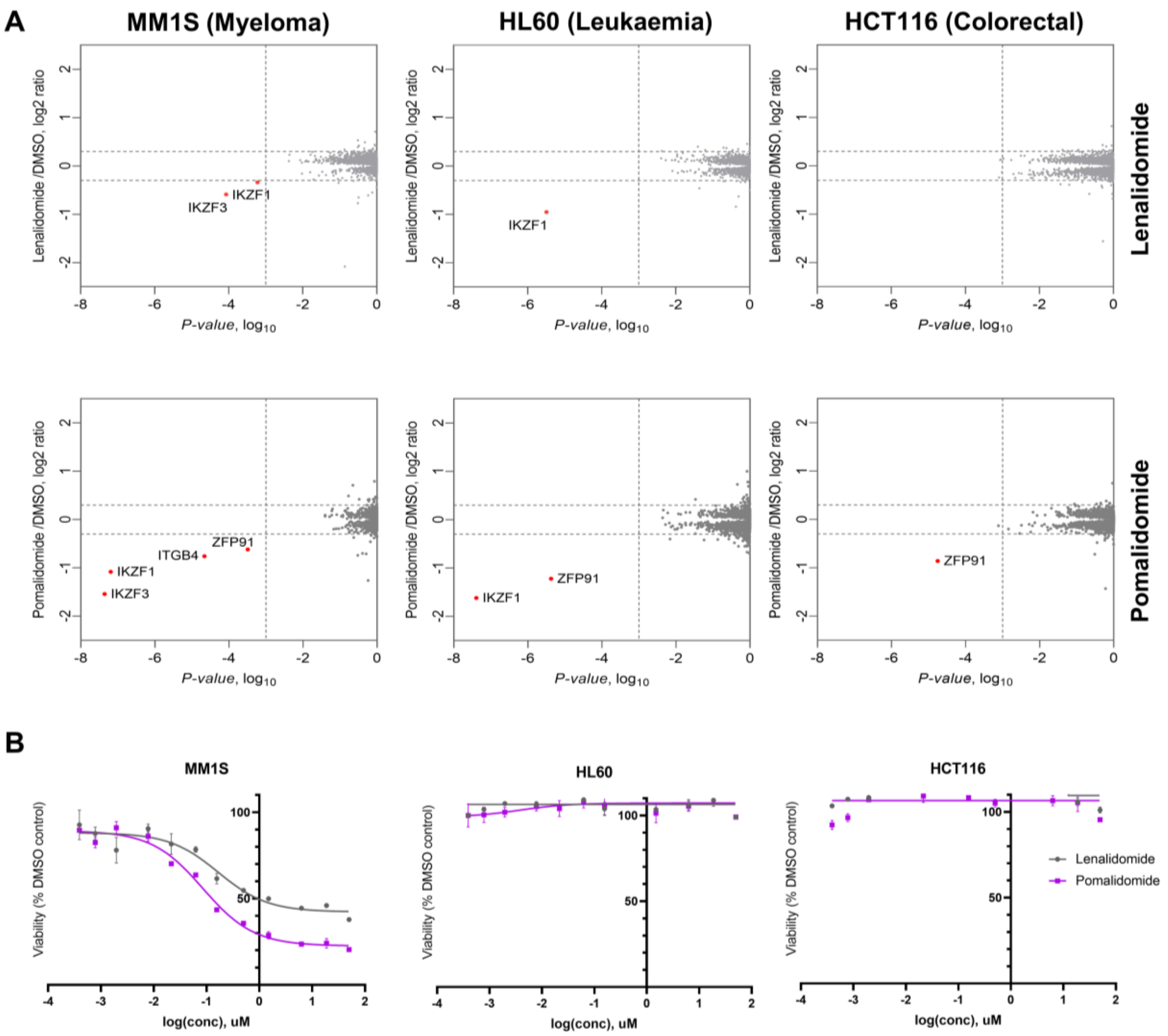

Figure S3. Proteomic profiles and cellular activities of lenalidomide and pomalidomide. A) Volcano plots highlighting lenalidomide- and pomalidomide-induced degradation targets in MM1S, HL60 and HCT116. B) Dose-response curves for MM1S, HL60 and HCT116 treated with lenalidomide and pomalidomide.

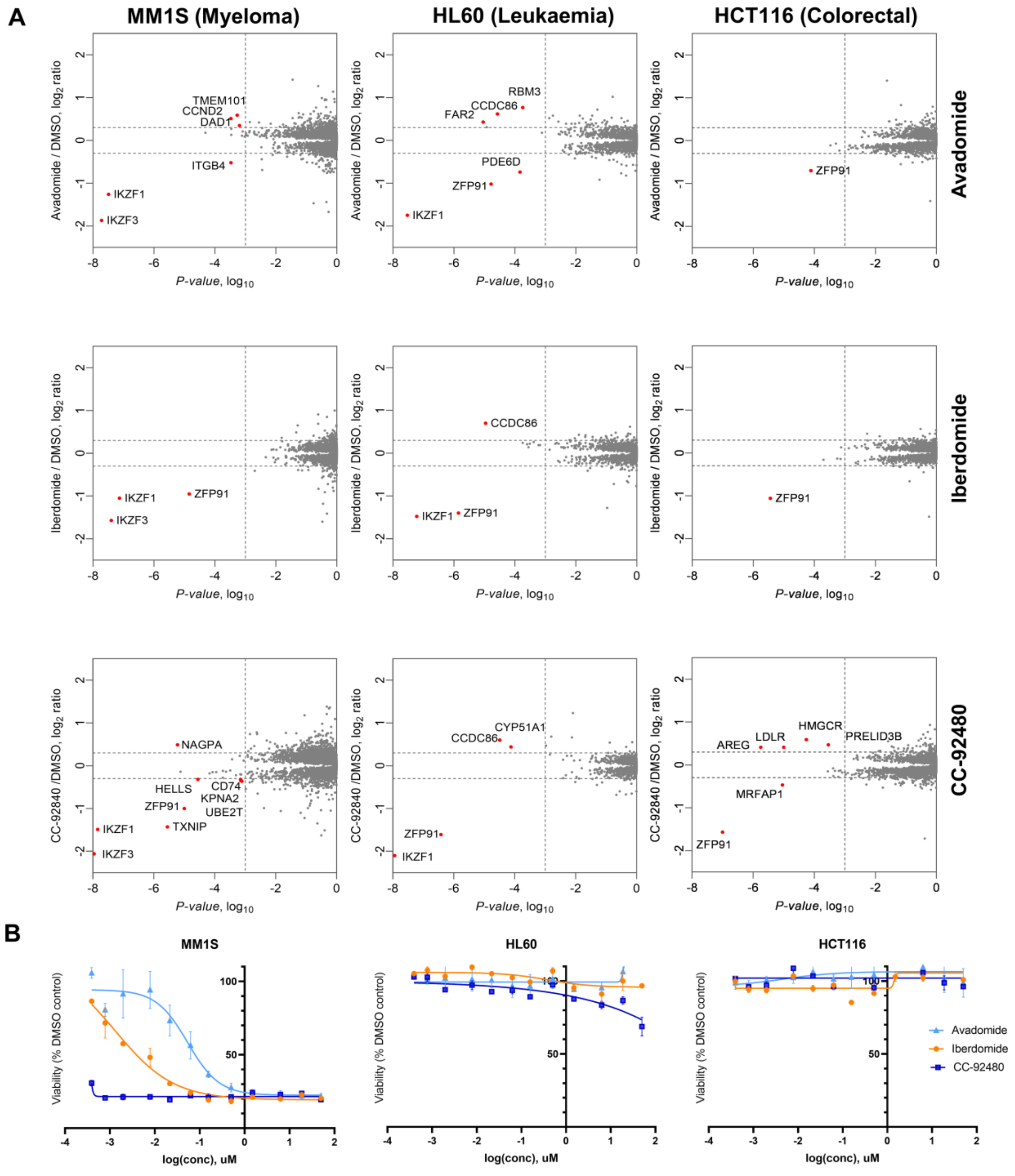

Figure S4. Proteomic profiles and cellular activities of avadomide, iberdomide and CC-92480. A) Volcano plots highlighting avadomide-, iberdomide- and CC-92480-induced targets in MM1S, HL60 and HCT116. B) Dose-response curves for MM1S, HL60 and HCT116 treated with avadomide, iberdomide and CC-92480.

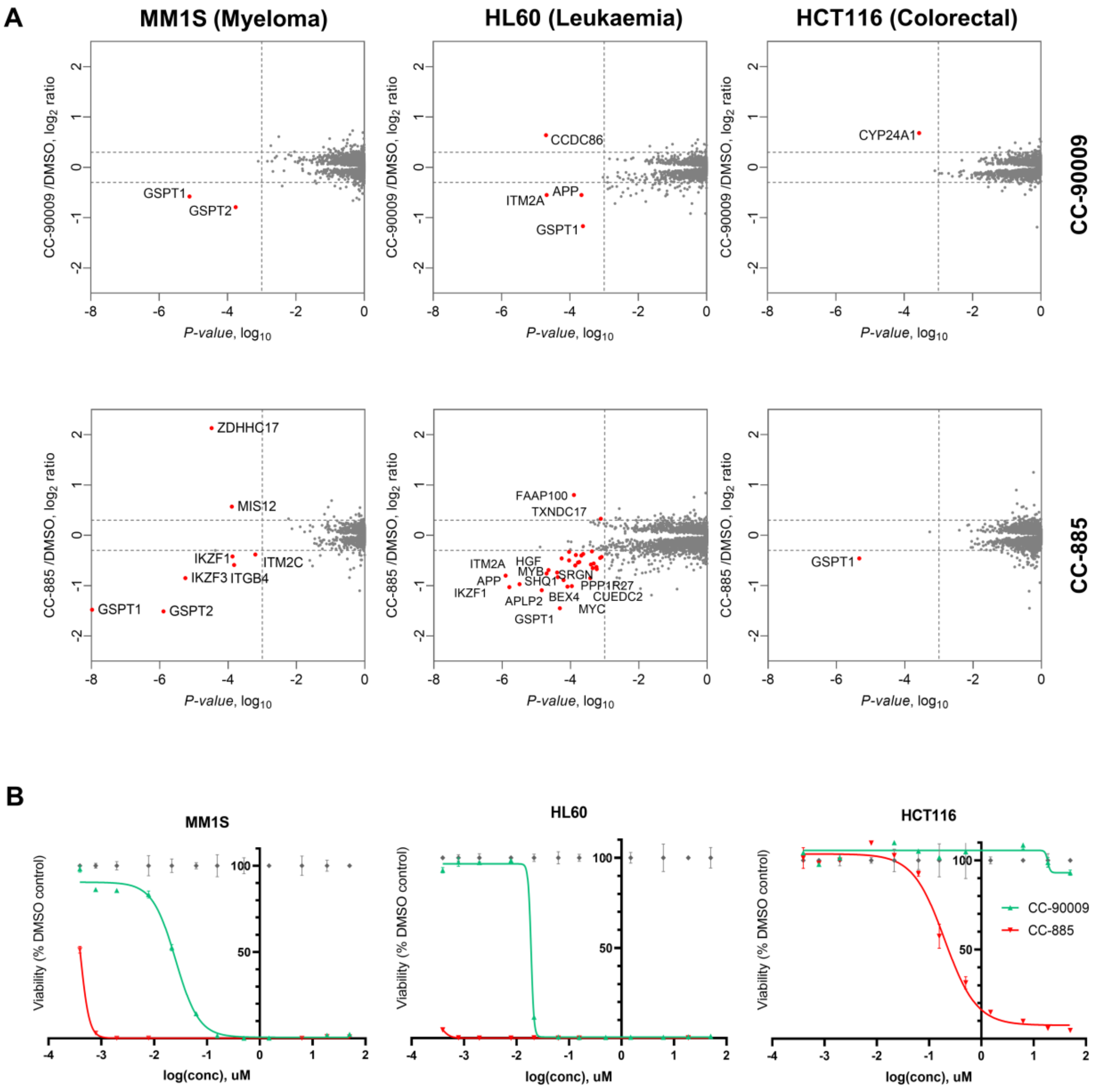

Figure S5. Proteomic profiles and cellular activities of CC-90009 and CC-885. A) Volcano plots highlighting CC-90009 and CC-885-induced targets in MM1S, HL60 and HCT116. B) Dose-response curves for MM1S, HL60 and HCT116 treated with CC-90009 and CC-885.

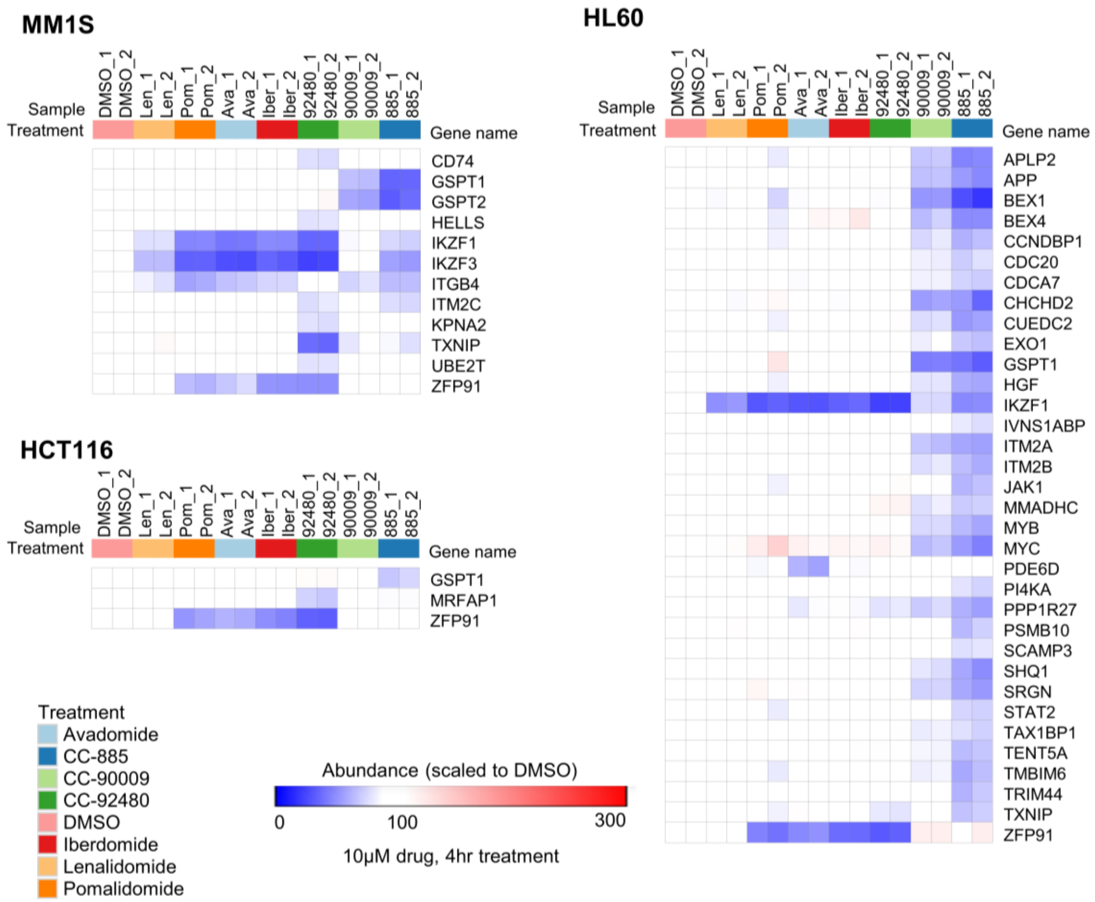

Figure S6. Protein degradation profiles of IMiDs/CELMoDs in three different cell lines. Heatmaps showing the relative protein abundance profiles of the significantly degraded proteins.

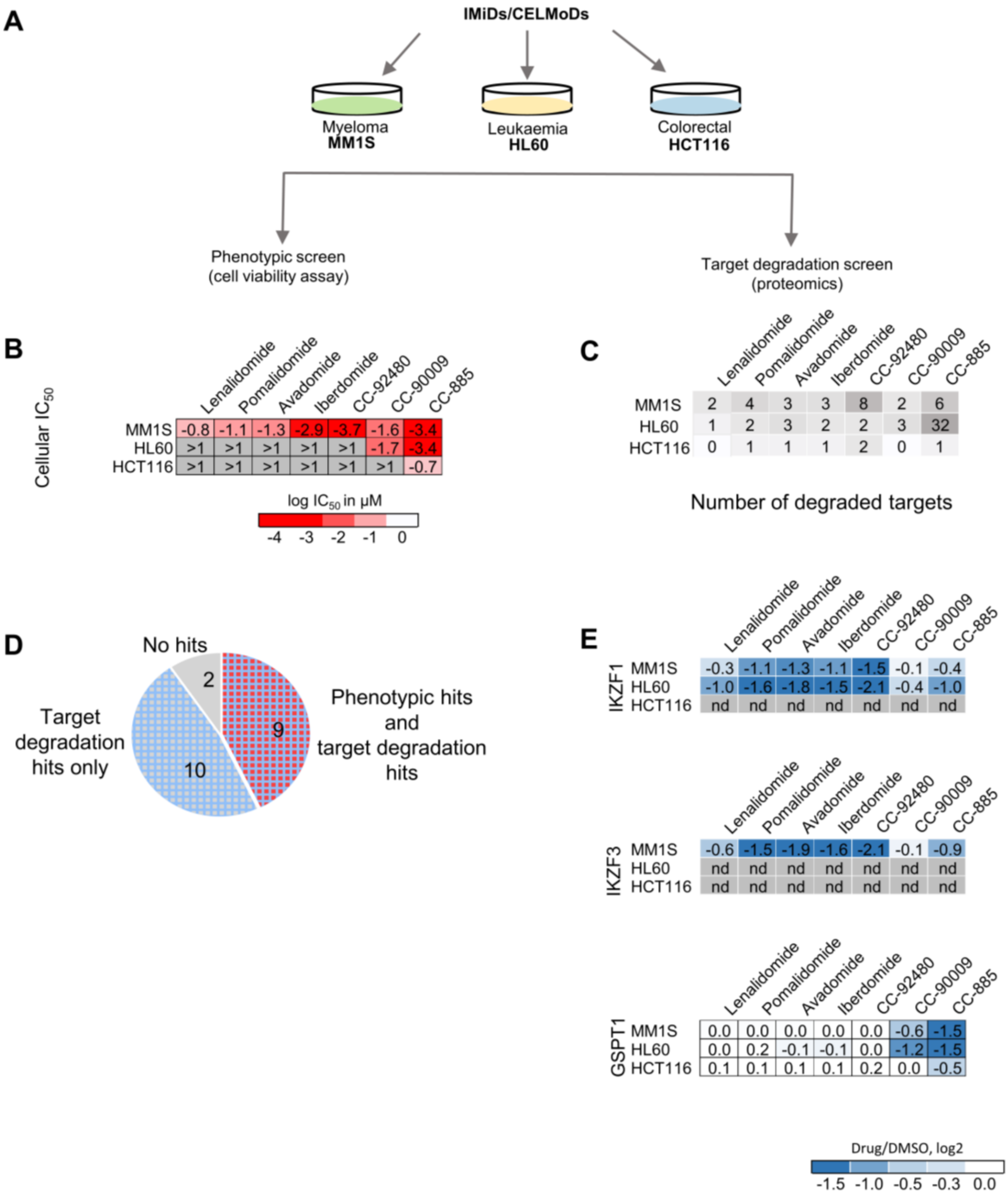

Figure S7. Summary of degradation targets and drug activity. A) Experimental design of phenotypic and target degradation screen. B) Heatmap representation of active compounds (red, IC50<10µM and max kill >50%) at 5-day treatment. C) Number of degraded proteins per compound and cell line in two biological replicates (T-test using log2 drug/DMSO < -0.32 and p < 0.001). D) Pie charts showing the fraction of hit compounds from phenotypic and target degradation screening. E) Heatmaps displaying log2 fold-change of known IMiD/CELMoD targets in three cell lines. Cell lines with undetectable protein levels are labelled as grey (not detected, nd).

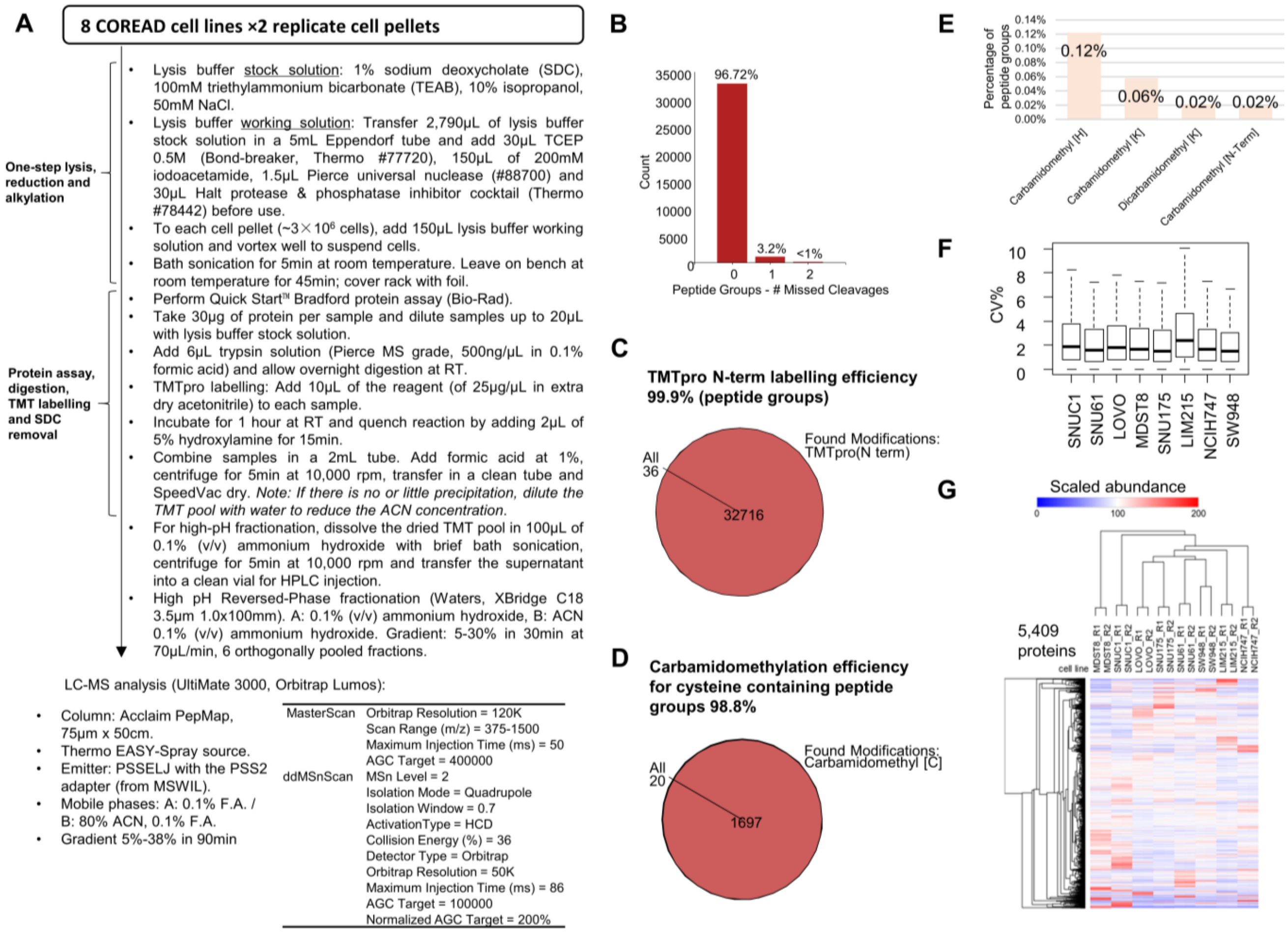

Figure S8. Evaluation of further improvements in the SimPLIT workflow. A) Detailed workflow for the in-lysis reduction/alkylation, TMTpro experiment comparing 8 different COREAD cell lines in replicate cell pellets. B) Bar plot of trypsin missed-cleavages. C) Venn diagram of all peptides and peptides with N-term TMTpro modification. D) Venn diagram of all cysteine containing peptides and peptides with carbamidomethylation at C. E) Bar plot of carbamidomethylation side reaction frequencies at peptide groups. F) Box plots of protein coefficient of variation (%) between replicate cell pellets per cell line. G) Heatmap of scaled protein abundances (row mean=100) for all quantified proteins.

Western blotting

Western blot. Cells were treated with compounds and incubated as indicated in the Figures and legends. Collected cell pellets were solubilised in SDS lysis buffer (1.5% SDS, 100 mM NaCl, 20 mM TrisHCl pH 6.8) sonicated and centrifuged at 13,000 × g for 10 min. Protein quantities were estimated with the Pierce BCA protein assay (ThermoFisher Scientific, IL, USA). Protein samples (5 or 10 µg) were warmed at 37 °C for 20 min with a Laemmli sample buffer. Protein samples were separated on 10

or 15% SDS-PAGE gels (Bio-rad) and transferred onto PVDF membranes using wet electroblotting. Membranes were blocked with LiCor blocking solution (LiCor), and incubated with primary antibodies overnight, followed by three washes in LiCor blocking solution and incubation with secondary antibodies for 1 h in the dark. After three final washes, the membranes were imaged on a LiCor fluorescent imaging station (LiCor). The raw images are shown below.

Related to Figure 5C

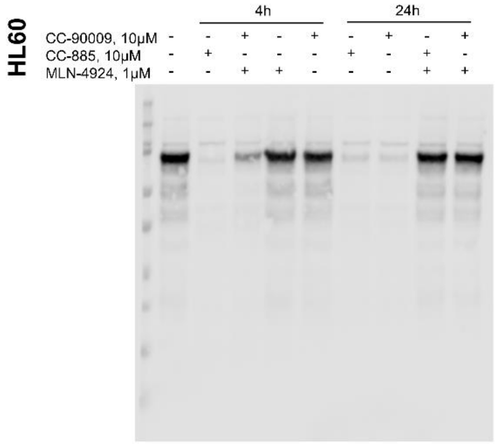

GSPT1

Related to Figure 5C

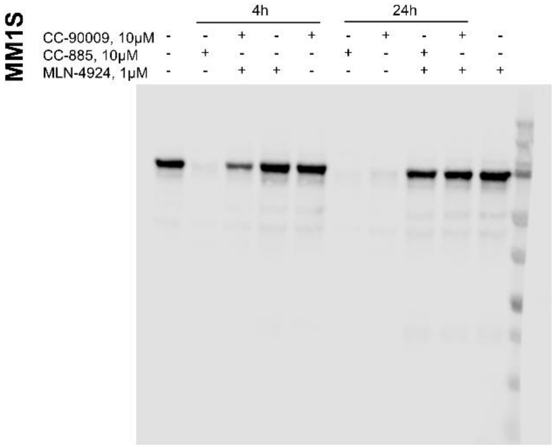

GSPT1

Related to Figure 5C

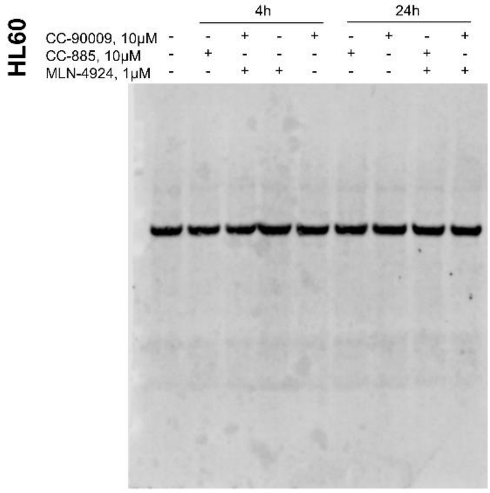

Actin

Related to Figure 5C

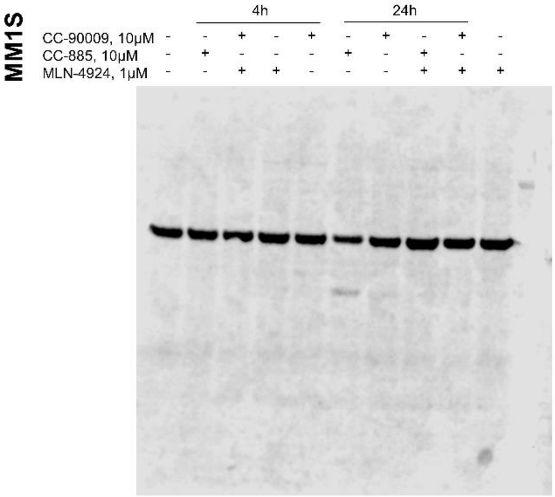

Actin

Related to Figure 5C

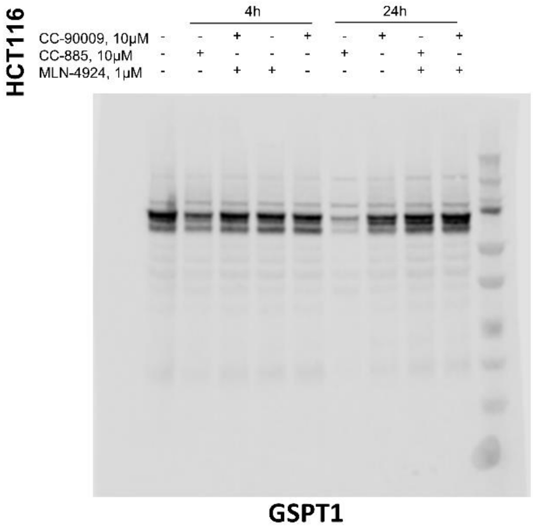

Related to Figure 5D

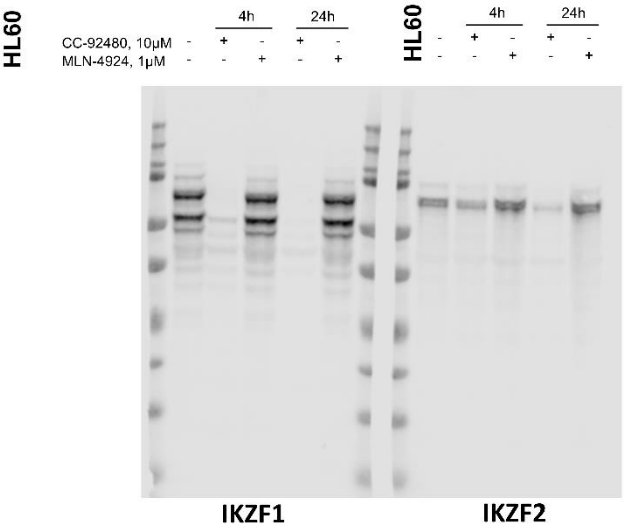

Related to Figure 5C

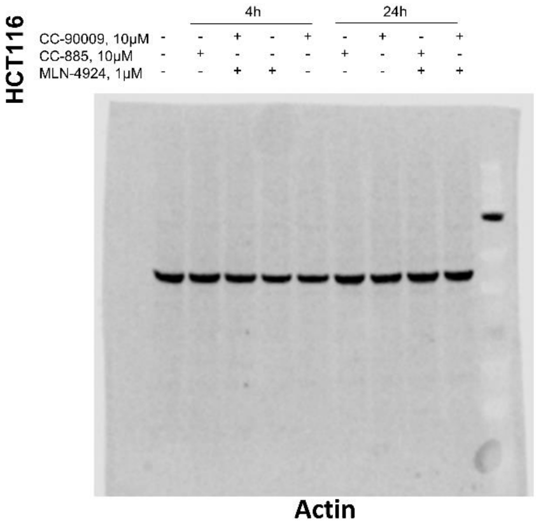

Related to Figure 5D

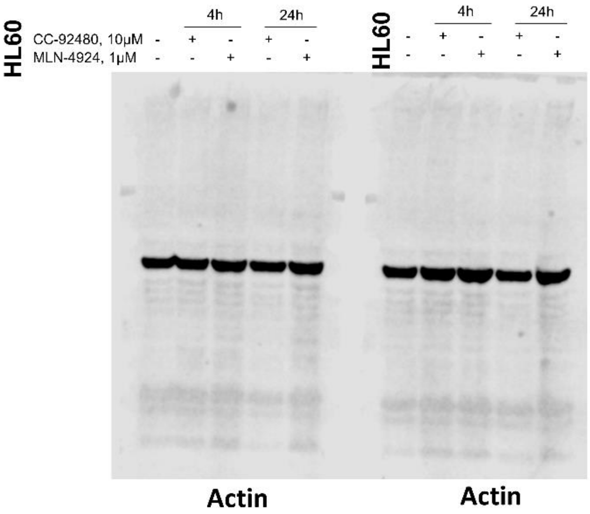

Supplementary table captions

Table S1. Detailed flowcharts for the different methods and indicative timings.

Table S2. Protein abundance data from the method comparison experiment measured by TMTpro-SPS-MS3.

Table S3. Protein abundance data for 45 COREAD cell lines and three pooled controls measured by TMTpro-HRMS^2 using the SimPLIT method.

Table S4. Protein abundance data for drug-treated cell lines (MM1S, HL60, HCT116) measured by TMTpro-HRMS^2 using the SimPLIT method.

Table S5. Differentially regulated proteins in at least ~20% of the measured COREAD cell lines and GO-BP enrichment analysis by STRING [1].

Table S6. MSI-high associated proteins in colorectal cancer cell lines.

Table S7. Protein abundance data from the in-lysis reduction/alkylation TMTpro16plex experiment.

1. Szklarczyk, D.; Gable, A. L.; Nastou, K. C.; Lyon, D.; Kirsch, R.; Pyysalo, S.; Doncheva, N. T.; Legeay, M.; Fang, T.; Bork, P.; Jensen, L. J.; von Mering, C., The STRING database in 2021: customizable protein-protein networks, and functional characterization of user-uploaded gene/measurement sets. *Nucleic Acids Res* **2021,** *49* (D1), D605-D612.
